# Designed Ankyrin Repeat Proteins as Actin Labels of Distinct Cytoskeletal Structures in Living Cells

**DOI:** 10.1021/acsnano.3c12265

**Published:** 2024-03-15

**Authors:** Julia R. Ivanova, Amelie S. Benk, Jonas V. Schaefer, Birgit Dreier, Leon O. Hermann, Andreas Plückthun, Dimitris Missirlis, Joachim P. Spatz

**Affiliations:** ∇ Department of Cellular Biophysics, Max Planck Institute for Medical Research, Jahnstrasse 29, D-69120 Heidelberg, Germany; ‡ Department of Biochemistry, University of Zurich, Winterthurerstrasse 190, 8057 Zurich, Switzerland; § Institute for Molecular Systems Engineering and Advanced Materials, Heidelberg University, INF 225, D-69120 Heidelberg, Germany; ∥ Heidelberg University, Faculty of Biosciences, 69120 Heidelberg, Germany; ⊥ Max Planck School Matter to Life, Jahnstrasse 29, 69120 Heidelberg, Germany; # CSL Behring AG, 3014 Bern, Switzerland

**Keywords:** Actin labels, Filopodia, Live-cell microscopy, Cytoskeleton dynamics, Retrograde flow

## Abstract

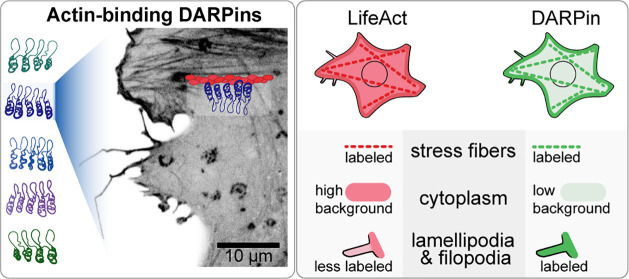

The orchestrated assembly of actin and actin-binding proteins into cytoskeletal structures coordinates cell morphology changes during migration, cytokinesis, and adaptation to external stimuli. The accurate and unbiased visualization of the diverse actin assemblies within cells is an ongoing challenge. We describe here the identification and use of designed ankyrin repeat proteins (DARPins) as synthetic actin binders. Actin-binding DARPins were identified through ribosome display and validated biochemically. When introduced or expressed inside living cells, fluorescently labeled DARPins accumulated at actin filaments, validated through phalloidin colocalization on fixed cells. Nevertheless, different DARPins displayed different actin labeling patterns: some DARPins labeled efficiently dynamic structures, such as filopodia, lamellipodia, and blebs, while others accumulated primarily in stress fibers. This differential intracellular distribution correlated with DARPin–actin binding kinetics, as measured by fluorescence recovery after photobleaching experiments. Moreover, the rapid arrest of actin dynamics induced by pharmacological treatment led to the fast relocalization of DARPins. Our data support the hypothesis that the localization of actin probes depends on the inherent dynamic movement of the actin cytoskeleton. Compared to the widely used LifeAct probe, one DARPin exhibited enhanced signal-to-background ratio while retaining a similar ability to label stress fibers. In summary, we propose DARPins as promising actin-binding proteins for labeling or manipulation in living cells.

## Introduction

Actin is a highly conserved protein, ubiquitously expressed in eukaryotic cells and typically the most abundant intracellular protein. Actin transitions from its monomeric state (globular or G-actin) to its polymerized state (filamentous or F-actin) in a process that is controlled by ATP hydrolysis and physical forces, with a variety of actin-binding proteins (ABPs) serving to nucleate, cap, or activate the monomers.^1,2^ The actin cytoskeleton fulfills fundamental functions of stabilizing cell shape, mechanically probing the cell environment and enabling cell motility.^3,4^ In addition, the F-/G-actin ratio is an important regulator of cell metabolism, affecting related signaling pathways and dictating cell decisions.^5^

Beyond being a structural element, the actin cytoskeleton provides the template for active force generation through myosin-driven contraction of antiparallel actin bundles.^6^ The formation of these bundles, called stress fibers, requires integrin-mediated adhesion to the extracellular environment through focal adhesions (FAs).^7^ At FAs, the local assembly of actin polymerization is triggered and actin filaments are thereafter cross-linked with the help of proteins such as α-actinin.^8^ Stress fibers are categorized in three major subtypes: (1) ventral stress fibers that are connected to the substrate at both ends via FAs, (2) dorsal stress fibers that begin at FAs and grow toward the dorsal region of the cell, and (3) transverse arcs that are curved parallel to the cell edge. Transverse arcs are not anchored to the extracellular matrix but are associated with dorsal stress fibers.^4^

Actin additionally assembles in diverse functional structures, under the control of specific ABPs. For example, at the cell edge, actin filaments assemble into filopodia, which are parallel bundles cross-linked by fascin that explore the extracellular space.^9^ Alternatively, branched actin networks can form lamellipodia at the protruding edge of cells, under the control of the Arp2/3 complex.^10,11^ Actin polymerization occurs at the plasma membrane, pushing the plasma membrane forward and concurrently traveling toward the cell interior due to actomyosin contractility, in what is known as actin retrograde flow.^12^ Coupling of the actin retrograde flow at adhesion clusters to integrins through multi-protein complexes results in force transmission to the cell exterior and enables mechanosensing.^13,14^ Overall, the concerted action of the aforementioned actin networks and ABPs enables a cell to probe its physical microenvironment and navigate in it.

In order to gain a detailed understanding of actin-mediated functions and effects, the reliable and accurate labeling of the actin cytoskeleton in living cells is required. Hence, much effort has been, and is being, devoted to develop probes that will efficiently report on actin location and dynamics, with minimal effects on physiological actin (de)polymerization and/or interactions with ABPs.^15−17^ Despite the development and wide use of many such probes, significant challenges remain. For example, toxin-derived probes based on phalloidin or jasplakinolide (SiR-actin) affect actin polymerization and hinder depolymerization.^18^ Furthermore, actin labels may show a biased distribution due to competition with endogenous actin-binding proteins, diffusion barriers, or actin turnover and, thus, may not accurately represent the actual actin cytoskeleton.^19−22^ SiR-actin, the protein-derived probe F-tractin, and utrophin domains insufficiently label highly dynamic structures such as filopodia and lamellipodia. Life-Act overcomes this issue in respect to its ability to accumulate in lamellipodia, but it insufficiently labels filopodia, the lamella, and the cytokinesis ring.^15,19,20^ Hence, the need for improved actin labels that efficiently label all actin structures remains.

**Figure 1:**
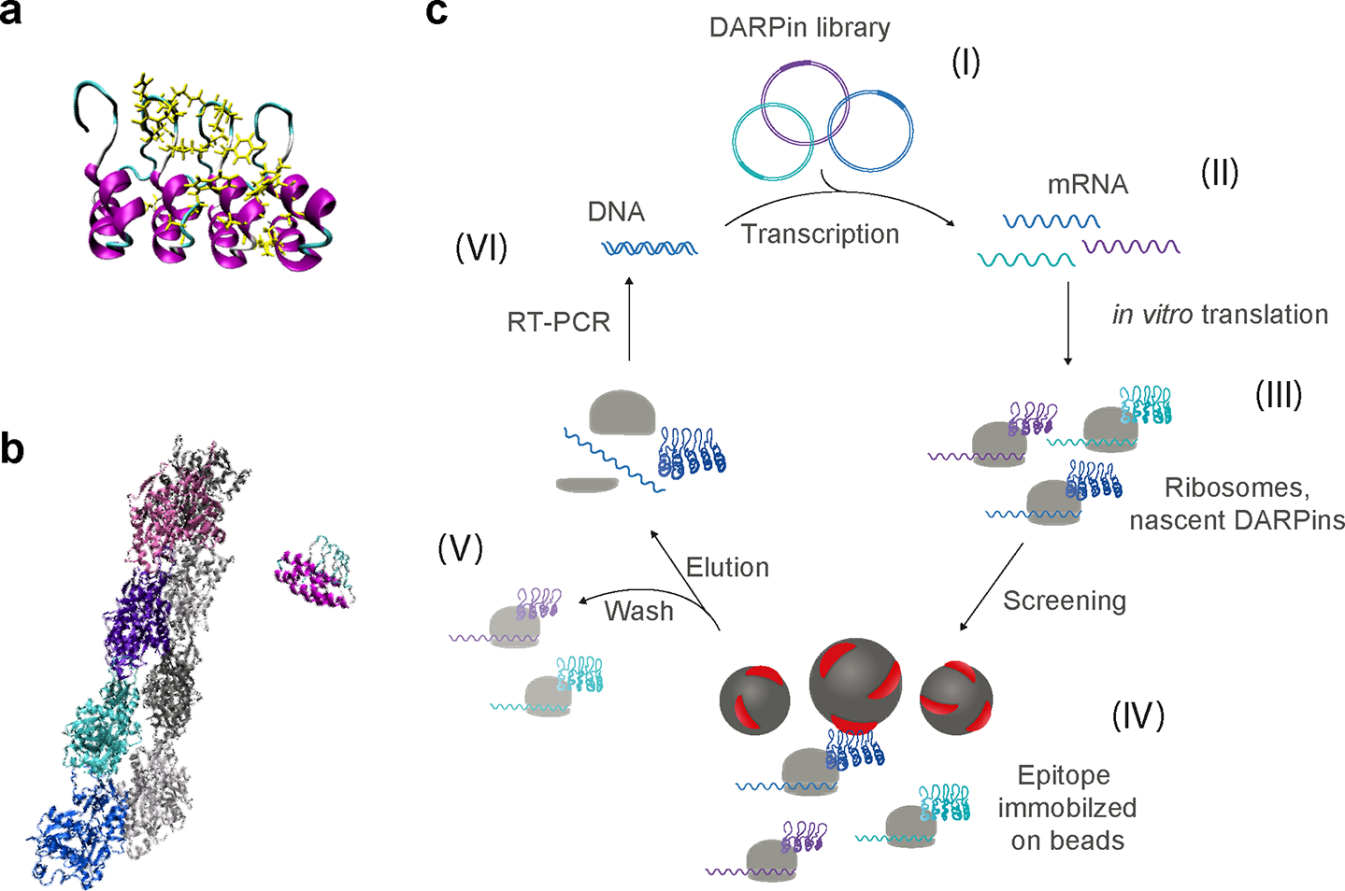
**DARPin structure and screening process. (a)** Structure prediction of an actin-binding DARPin (1784_A7) from the online software tool I-Tasser^30^ showing the four ankyrin repeat units, each one with two antiparallel alpha-helices (purple) linked via flexible loops (cyan). The specificity for actin binding is created by randomizing a subset of amino acids in the DARPin sequence (highlighted yellow). **(b)** The DARPin next to a helical actin minifilament composed of eight actin monomers (PDB: 6BNO) is shown for scale comparison. For the sake of clarity, the actin monomers have different colors. **(c)** Schematic of the DARPin selection workflow by ribosome display. (I) A DNA-based library of DARPins was (II) transcribed *in vitro* to mRNA. (III) During *in vitro* translation, a lack of stop codon in a spacer at the end of the DARPin sequence allows the DARPin to fully emerge from the ribosome, as well as ribosome, mRNA, and protein complex, to remain intact while the DARPin is folding into its tertiary structure. (IV) The complexes presenting the DARPins are screened in two independent selections for binding against immobilized G-actin or F-actin, respectively. (V) Unbound DARPin complexes were washed off with increasing stringency, and finally the binders were eluted together with the corresponding mRNA. (VI) Reverse transcription PCR was performed to recover the genetic information of the binders. In total, four selection rounds with increasing stringency in the washing steps were performed. The enriched binders are then expressed in *E. coli* and further characterized, first in crude extracts and then as purified proteins.

In this study, we explored the possibility of using designed ankyrin repeat proteins (DARPins) as actin-binding probes inside living cells. DARPins are cysteine-free, low molecular weight, synthetic proteins (12–20 kDa), that are about 1/4 to half the size of globular actin (42 kDa; Figure 1a,b). Their scaffold is made up of two to three central ankyrin repeat motifs with a helix-turn-helix conformation, flanked by N- and C-terminal capping units (Figure 1a). By randomizing exposed amino acids from the central repeats and/or the capping units and applying suitable selection and evolution strategies (for details, see Materials and Methods), binding specificities to essentially any target can be achieved, and affinities for individual DARPins can be tuned.^23^ The lack of cysteines in their sequence allows correct folding in the reducing environment of the cytoplasm and thus renders DARPins—in contrast to antibodies—as promising candidates for intracellular applications. In past studies, DARPins have been expressed in the cytosol of mammalian cells to inhibit, e.g., c-Jun N-terminal kinases with isoform specificity,^24^ or to target extracellular signal-regulated kinase ERK,^25^ as well as other cellular targets.^23^ Furthermore, DARPins have been directly delivered to the cytosol, e.g., by a bacterial system to target Ras.^26^ Finally. they have been used as targeting agents on lentiviral, or adeno-associated viral (AAV) vectors^27^ and adenoviruses.^28,29^ In this work, we aimed to identify DARPins that bind to the actin cytoskeleton and explore their ability as intracellular actin labels.

## Results

### Selection and *In Vitro* Evaluation of Actin-Binding DARPins

To select for actin-binding DARPins, we performed two independent selections by ribosome display (Figure 1c).^31^ The libraries used and the adaptation to a semiautomated high-throughput system are described in the Materials and Methods. The first selection was performed against bead-immobilized prepolymerized F-actin and the second against monomeric G-actin from rabbit skeletal muscle. Rabbit skeletal muscle actin has a sequence homology to human skeletal muscle actin and human cytoplasmic actin of 100% and 93.33%, respectively (Figure S1).

Selected DARPin sequences were subcloned in bacterial expression vectors, and 380 crude extracts per selection were screened for actin binding by a qualitative enzyme-linked immunosorbent assay (ELISA) against surface-immobilized target (Figures S2A and S3A). Due to an unusually low number of hits in selection 1, these crude extracts were screened additionally by an in-solution homogeneous time-resolved fluorescence (HTRF) assay (Figure S2B). 12 and 123 positive clones from selection one and two, respectively, were identified. Subsequent Sanger sequencing of 40 positive clones and singularization of 4 double clones resulted in a final 27 unique DARPins, which were purified by immobilized metal ion affinity chromatography (IMAC). In the case of the second selection, DARPin binding to G-actin was further validated by a quantitative ELISA (Figure S3B).

The oligomeric state and susceptibility to aggregation of purified DARPin proteins was then probed by analytical size exclusion chromatography (Figure S4). 13/27 analyzed DARPins showed a clearly monomeric behavior (left column in Figure S4; e.g. DARPin 1784_A7), while 9/27 DARPins demonstrated a tendency to form dimers (middle column in Figure S4; e.g. DARPin 2356_E6). The remaining 5/27 DARPins aggregated (right column in Figure S4; e.g. DARPin 1784_G9) and were therefore excluded from further analysis.

In order to examine whether DARPins impact the actin polymerization process, an *in vitro* polymerization assay using pyrene-actin was performed^32,33^ (Figure S5). All analyzed DARPins enabled actin polymerization with minimal impact on polymerization kinetics; however, a significant reduction in the final F-actin levels was observed in the presence of 5 DARPins (DARPins 2357_B2, 2359_A11, 2359_C6, 2359_F1, and 2359_G12).

The ability of the selected DARPins to bind F-actin *in vitro* was next assessed by mixing purified, His-tagged DARPins with F-actin filaments and detecting bound DARPins via fluorescent anti-His-tag antibodies and confocal laser scanning microscopy (Figure S6). Among the 22 candidates, 7 DARPins clearly visualized F-actin filaments (highlighted in red boxes in Figure S6), 2 DARPins showed some fluorescence signal above background but did not efficiently stain F-actin (highlighted in blue boxes in Figure S6), and the other 13 DARPins did not show any signal above background under this experimental setup. Overall, from our *in vitro* screen, 22 actin-binding DARPins were identified, albeit with variable potential to stain purified F-actin.

### Microinjection of Actin-Binding DARPins in Living Cells

To introduce DARPins inside living cells, we initially selected the DARPin clone 1784_A7, which showed efficient *in vitro* labeling of actin filaments (Figure S6), and the control DARPin E3_5, which was not expected to exhibit any preferential accumulation. The fluorescent probe Atto488 or Cy5 was successfully linked to the C-terminus of the purified DARPins via sortase-mediated coupling.^34^ The labeling efficacy of Atto488 to DARPin 1784_A7 and Cy5 to DARPin E3_5 was 1.2:1 and 0.9:1, respectively, indicating quantitative labeling (Figure S7).

**Figure 2:**
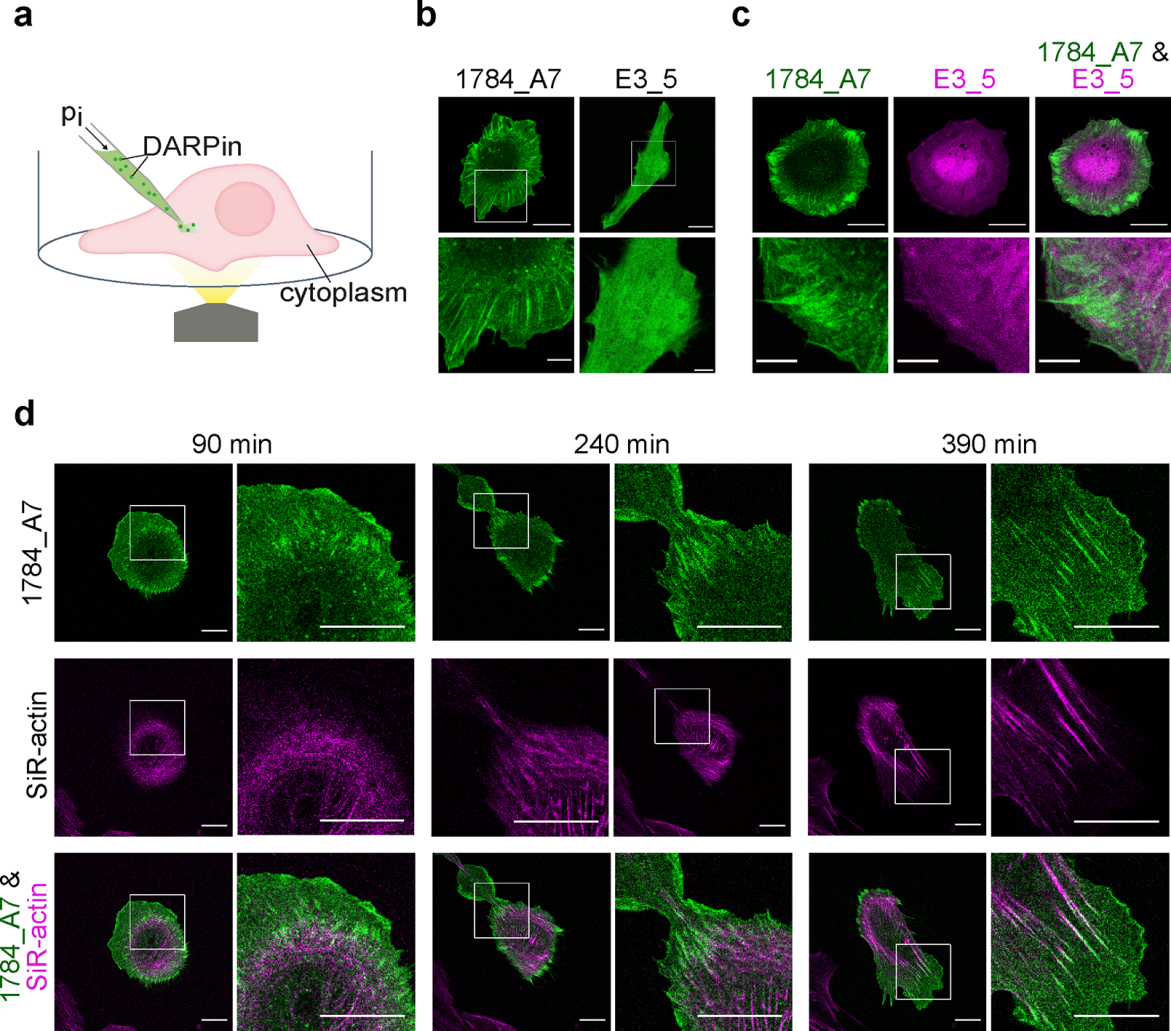
**Microinjection of DARPins in living cells**. **(a)** Schematic representation of the microinjection procedure. A microinjection needle filled with DARPin solution penetrates the cell membrane, and its contents are released in the cytoplasm. The volume and therefore the number of injected DARPin proteins depend on the injection pressure (pi) and duration of injection. **(b)** Confocal microscopy images of live pHDF cells injected with Atto488-labeled DARPin 1784_A7 or control DARPin. DARPin 1784_A7 localization was typical of F-actin, while the control DARPin was homogeneously distributed in the cytoplasm. Scale bars for whole-cell images: 20 μm; details: 5 μm. **(c)** Confocal microscopy images of a pHDF cell co-injected with Atto488-labeled DARPin 1784_A7 (green) and Cy5-labeled control DARPin (magenta). Scale bars for whole-cell images: 20 μm; details: 5 μm. **(d)** Confocal microscopy images of a pHDF cell injected with DARPin 1784_A7 and labeled with SiR-actin at the cell-substrate plane acquired during time-lapse imaging. SiR-actin stained prominently stress fibers, while DARPin 1784_A7 labeled preferentially the ends of stress fibers and cortical actin structures. Scale bars: 20 μm.

Single DARPins were introduced in primary human fibroblasts (pHDFs) via microinjection, a technique that allows the direct entry, and immediate monitoring, of DARPins in the cytoplasm (Figure 2a). Confocal fluorescence microscopy of live cells revealed that DARPin 1784_A7 accumulated at the cell edge in regions that resemble lamellipodia as well as in elongated clusters and fibers oriented perpendicularly to the edge that appeared to be focal adhesions and stress fibers, respectively (Figure 2b). In contrast, the nonbinder DARPin was homogeneously distributed in the cytoplasm and nucleus, as expected (Figure 2b). When both DARPins were co-injected in the same cell, their localization patterns were distinct and corresponded to the ones observed for cells injected with single DARPins (Figure 2c). Microinjection of DARPin clone 1784_A7 in mouse kidney fibroblasts and U2OS cells resulted in a similar intracellular distribution, characteristic of the actin cytoskeleton pattern, indicating the general applicability in different cell types (Figure S8).

The localization of injected actin-binding DARPin was compared in living cells to SiR-actin, a widely used cell-permeable F-actin dye (Figure 2d and Movie S1). SiR-actin is a derivative of the F-actin-stabilizing drug jasplakinolide, which has a low dissociation constant in the nM range^17,35^ and thus is often considered to stain mature actin structures.^36^ Accordingly, SiR-actin predominantly labeled stress fibers in the cell body and was largely excluded from freshly polymerized actin in the lamellipodia (Figure 2d). The actin-binding DARPin, on the other hand, accumulated at the leading cell edge and at the ends of stress fibers, where it overlapped with SiR-actin. Overall, these findings indicate that DARPin 1784_A7 binds and labels the actin cytoskeleton of living cells.

### Differential Labeling of Intracellular Actin Structures by DARPins

Having validated the ability of a DARPin clone to label the actin cytoskeleton in living cells, we set out to examine the labeling preferences and thus localization of the other actin-binding DARPin clones identified in our *in vitro* screen. Since microinjection is a time-consuming and low throughput method, DARPins were subcloned in a mammalian vector system, adding a C-terminal mEGFP (monomeric enhanced green fluorescent protein), and were transiently transfected inside U2OS cells. We tested 16 out of the 22 DARPin candidates, based on a preliminary screen in mouse kidney fibroblasts (a list of the DARPin sequences, along with that of DARPin E3_5, an unselected member of library N3C that does not bind actin,^37^ is presented in Table S1). We selected the osteosarcoma U2OS cell line since it is commonly used in actin cytoskeleton and cell motility studies^4,38^ and showed more efficient transfection compared to the fibroblasts. The transfection efficiency using an optimized protocol with lipofectamine 3000 for the tested DARPins was around 50%, with similar expression levels for the different clones (Figure S9).

Confocal microscopy images of live cells were examined to evaluate the localization and actin-binding potential of mEGFP-DARPins. Cells with an intermediate expression level were selected by visual examination for all analyses. From the 16 tested DARPins, 11 appeared to label F-actin structures, albeit with pronounced differences in labeling patterns and signal-to-background ratio (Figure S10; highlighted in blue). For example, DARPins 1784_A7 and 1784_G3.3 labeled what appear to be lamellipodia and stress fibers, while DARPins 2356_E5, 2356_F1, and 2358_A11 accumulated preferentially at stress fibers and lamella (Figure S10). The remaining 5/16 DARPins exhibited homogeneous cytoplasmic fluorescence suggesting inefficient actin labeling. Of note, the intracellular distribution of microinjected Atto488-labeled DARPin 1784_A7 was similar to that of mEGFP-DARPin 1784_A7, indicating correct folding of the DARPin upon translation in the reducing environment of the cytoplasm and that C-terminal appending of the relatively bulky mEGFP via a flexible linker did not hinder actin binding. A qualitative assessment for the intracellular distribution of the tested DARPins is presented in the Table S2 .

**Figure 3:**
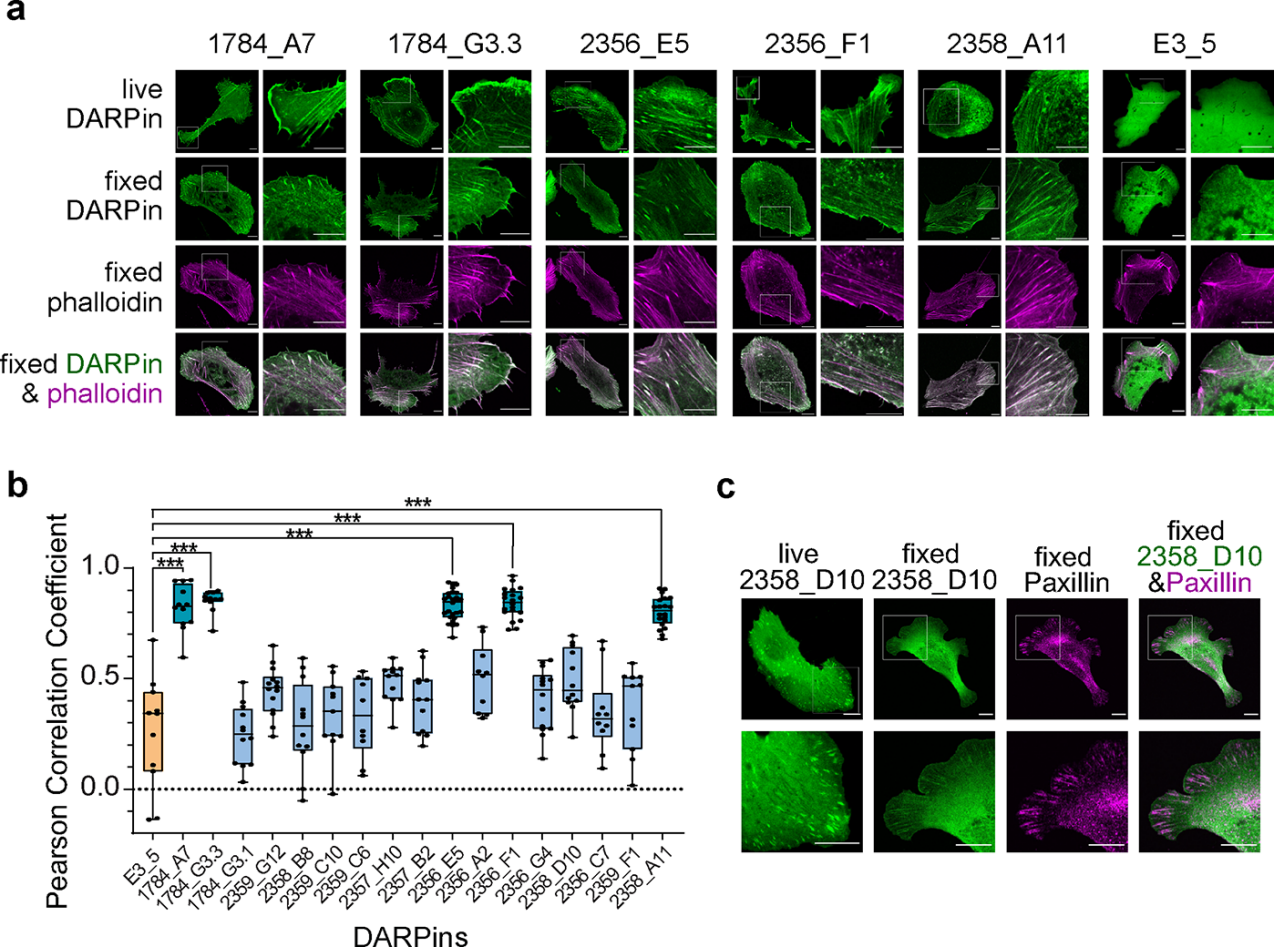
**DARPin colocalization with F-actin. (a)** Confocal images of live and fixed U2OS cells expressing different mEGFP-DARPins. In fixed cells, F-actin was additionally labeled with phalloidin. **(b)** Pearson’s correlation coefficient (PCC) values calculated from images of fixed, DARPin-expressing, and phalloidin-labeled U2OS cells. Each data point represents the PCC value for one cell. A minimum of ten cells from two independent experiments were analyzed. The box extends from the 25th to 75th percentile, the whiskers contain all the values, and the line represents the median value. Data were compared with a Welch ANOVA test: only PCC values that differ significantly from the control are shown (*P* < 0.005 (***)). **(c)** Confocal images of live and fixed U2OS cells expressing DARPin 2358_D10. Paxillin was immunolabeled in fixed cells and compared to the localization of DARPin 2358_D10. Scale bars:10 μm.

In order to validate and quantify the colocalization of DARPins with F-actin, transfected cells were fixed and labeled with the *bona fide* F-actin stain phalloidin and the Pearson’s correlation coefficient (PCC) was calculated (Figure 3a). The PCC values for DARPins 1784_A7, 1784_G3.3, 2356_E5, 2356_F1, and 2358_A11 were close to 1.0, demonstrating high colocalization with phalloidin and low background signal, while the rest of the DARPins exhibited PCC values similar to that of the control DARPin E3_5 (Figure 3b). For example, DARPin 2358_D10, which clustered in structures resembling FAs (Figure 3c), had a low PCC value, most likely due to high background cytoplasmic fluorescence. At the same time, DARPin 2358_D10 showed high colocalization with the FA marker paxillin, suggesting that it accumulates to actin at FAs, but not other actin structures (Figure 3c). In summary, we identified DARPins that can bind and stain F-actin in living cells, albeit with different labeling patterns.

### DARPin 1784_A7, but Not 2356_E5, Stains Lamellipodia

**Figure 4:**
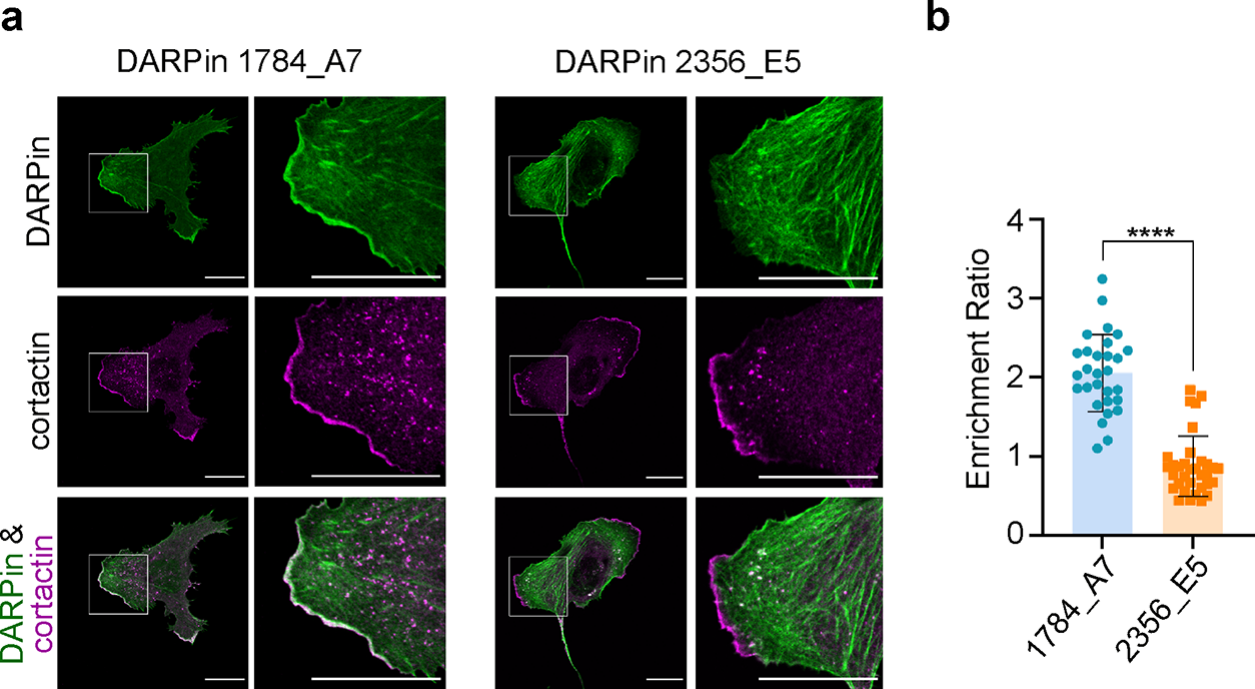
**Distinct localization of DARPins. (a)** Confocal microscopy images of mEGFP-DARPin-expressing U2OS cells (green) fixed 3 h after seeding and immunostained against the lamellipodia marker cortactin (magenta). Scale bars: 20 μm. **(b)** The enrichment ratio of DARPins in lamellipodia, calculated as the ratio of intensity in the lamellipodia to the intensity in the surrounding cytoplasm, was significantly higher for DARPin 1784_A7 compared to DARPin 2356_E5 (*p* < 0.0001 (****) in an unpaired two-tailed *t* test, *n* = 2 experiments, minimum 12 regions of interest per DARPin and experiment).

We next focused on the subset of DARPins that exhibited high colocalization coefficients with Phalloidin: DARPin 1784_A7, 1784_G3.3, 2356_E5, 2356_F1, and 2358_A11 (Figure 3b). These 5 DARPins labeled the three major types of stress fibers in U2OS seeded on glass substrates (Figure S11); however, we noticed that DARPins 1784_A7 and 1784_G3.3 additionally labeled efficiently lamellipodia, in contrast to DARPins 2356_E5, 2356_F1, and 2358_A11 (Figures 3a and 4a). On soft, silicone elastomers, on which U2OS fail to polarize and instead assemble multiple lamellipodia around their cell perimeter,^39^ the contrast between the two DARPins was more prominent (Movies S2 and S3). Super-resolution STED microscopy further supported these observations at higher resolution (Figure S12). In order to quantify DARPin accumulation in lamellipodia, the lamellipodia in protruding regions were delineated by cortactin immunostaining and the enrichment ratio (ER: fluorescence intensity within lamellipodia/fluorescence intensity in surrounding area) was calculated (Figure S13). The significantly higher ER values for DARPin 1784_A7 compared to DARPin 2356_E5 validated the microscopic observations (Figure 4b). Furthermore, we microinjected purified Cy5-labeled DARPin 1784_A7 in U2OS cells expressing DARPin 2356_E5 and observed distinct localization at the edge of the same cell, but not at stress fibers (Figure S14). The above result suggested that both DARPins can simultaneously bind F-actin, and it further highlighted how different DARPins accumulate at distinct actin structures within living cells.

### DARPin Localization Correlates with DARPin Actin Binding Kinetics

The distinct localization of different actin labels was previously related to differences in their F-actin binding kinetics.^19^ A fast turnover rate of the actin label, compared to the actin filament turnover rates and the speed of actin retrograde flow, is required to accurately label actin in lamellipodia.^21^ High binding affinity and low turnover would instead bias the localization of the probe toward the cell center due to retrograde flow.^40,19,21^ Hence, the actin binding kinetics of DARPins in living cells were assessed by fluorescence recovery after photobleaching (FRAP) in two different regions: ventral stress fibers and lamellipodia. For comparison, FRAP was also performed on SiR-actin-labeled cells and mCherry-LifeAct expressing cells.

**Figure 5:**
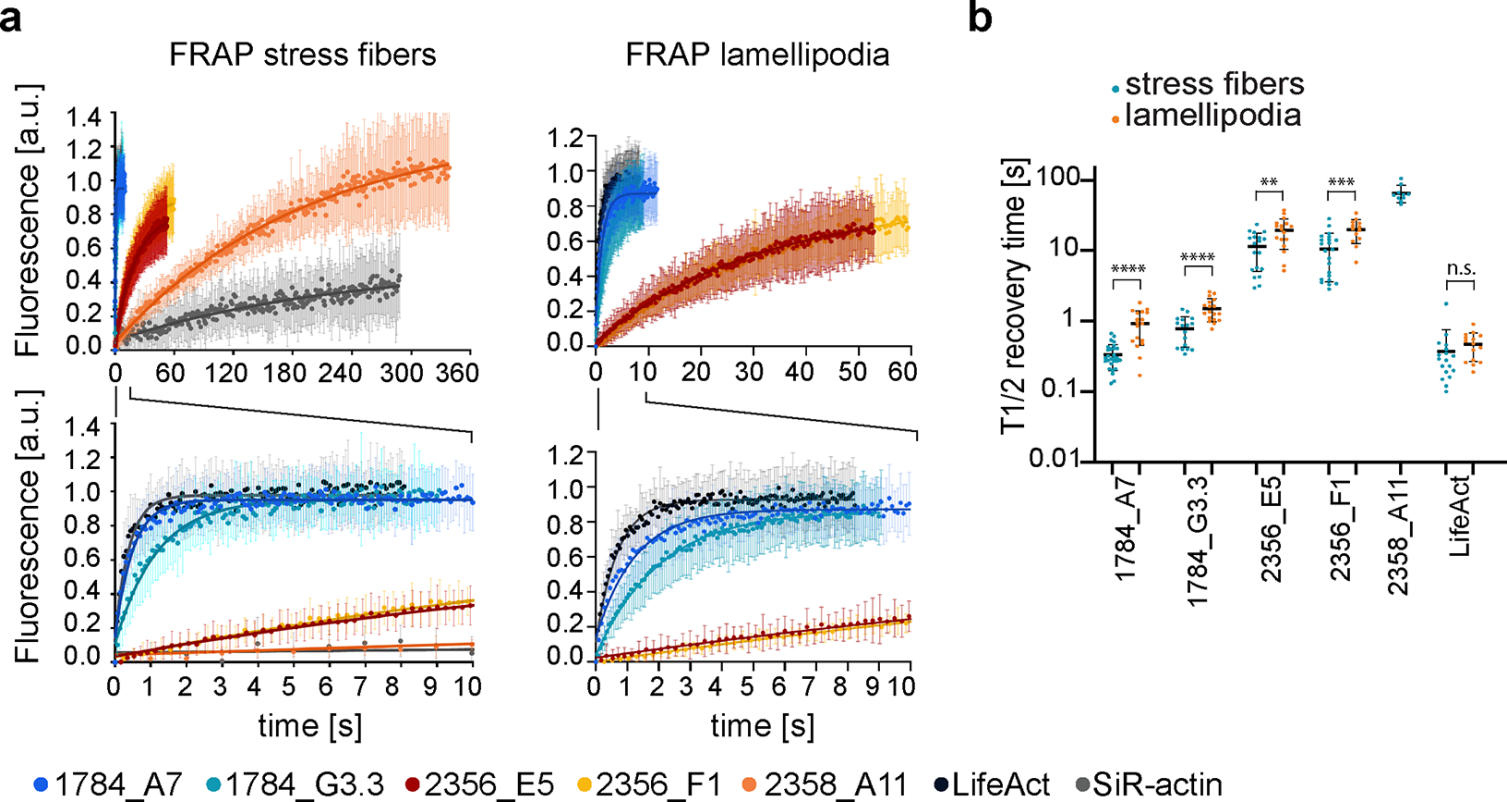
**DARPins exhibit varied actin binding kinetics in cells. (a)** Fluorescence recovery after photobleaching (FRAP) curves for mEGFP-DARPins, mCherry-LifeAct and SiR-actin performed on ventral stress fibers (left) and lamellipodia (right). The fluorescence was normalized to the intensity of the bleached regions before bleaching (=1) and directly after bleaching (=0). Zoomed-in regions are shown at the bottom. **(b)** The half recovery times for DARPins 1784_A7, 1784_G3.3, 2356_E5, 2356_F1, 2358_A11, and LifeAct calculated for FRAP on lamellipodia and ventral stress fibers. Each data point corresponds to a FRAP curve (*n* = 10–28) from *N* = 2–4 independent experiments. The line corresponds to the mean and error bars to the standard deviation. Half-recovery times for the same actin binder on ventral stress fibers were compared to lamellipodia using two-tailed unpaired *t* tests (LifeAct *p* = 0.38 (n.s.), 2356_F1 *p* = 0.0006 (***), 2356_E5 *p* = 0.0036 (**), 1784_G3.3 *p* < 0.0001 (****), 1784_A7 *p* < 0.0001 (****)).

The half-time (τ_1/2_) for fluorescence recovery at lamellipodia differed by more than 1 order of magnitude between the tested DARPins. DARPins 1784_A7 and 1784_G3.3 displayed fast turnover with τ_1/2_ values of 0.92 ± 0.45 and 1.51 ± 0.53 s, respectively (Figure 5a,b). These values are in the same order of magnitude as the τ_1/2_ = 0.47 ± 0.20 s calculated for LifeAct. In contrast, DARPins 2356_E5 and 2356_F1 exhibited longer half-times of 19.5 ± 8.8 and 20.0 ± 7.1 s, respectively. DARPin 2358_A11 and SiR-actin τ_1/2_ values were not determined for lamellipodia because their intensity was very low in these structures. Moreover, when a signal above background was present, fluorescence recovery was too slow with respect to lamellipodia moving out of the frame before substantial recovery.

On ventral stress fibers, the calculated τ_1/2_ for DARPins was slightly lower compared to the ones determined for lamellipodia (Figure 5a,b). The reason for this increase in DARPin turnover on stress fibers is not clear at present but is most likely due to the experimental procedure and steric effects in the highly dense lamellipodia. Lamellipodia are composed of a highly branched actin network sandwiched between the plasma membrane, and the size of actin-binding proteins has been shown to influence binding kinetics.^41^ On ventral stress fibers, we were able to perform FRAP on DARPin 2358_A11, as these were more stable than lamellipodia: the τ_1/2_ for DARPin 2358_A11 was determined to be 65 ± 17 s. SiR-actin exhibited even slower recovery kinetics with less than 50% recovered fluorescence within the 5 min of the experiment (Figure 5a). The mobile fractions for DARPins were high, with those of DARPin 1784_A7 and 1784_G3.3 close to 1, indicating transient binding and the absence of a population of stably bound DARPins (Figure 5a).

**Figure 6:**
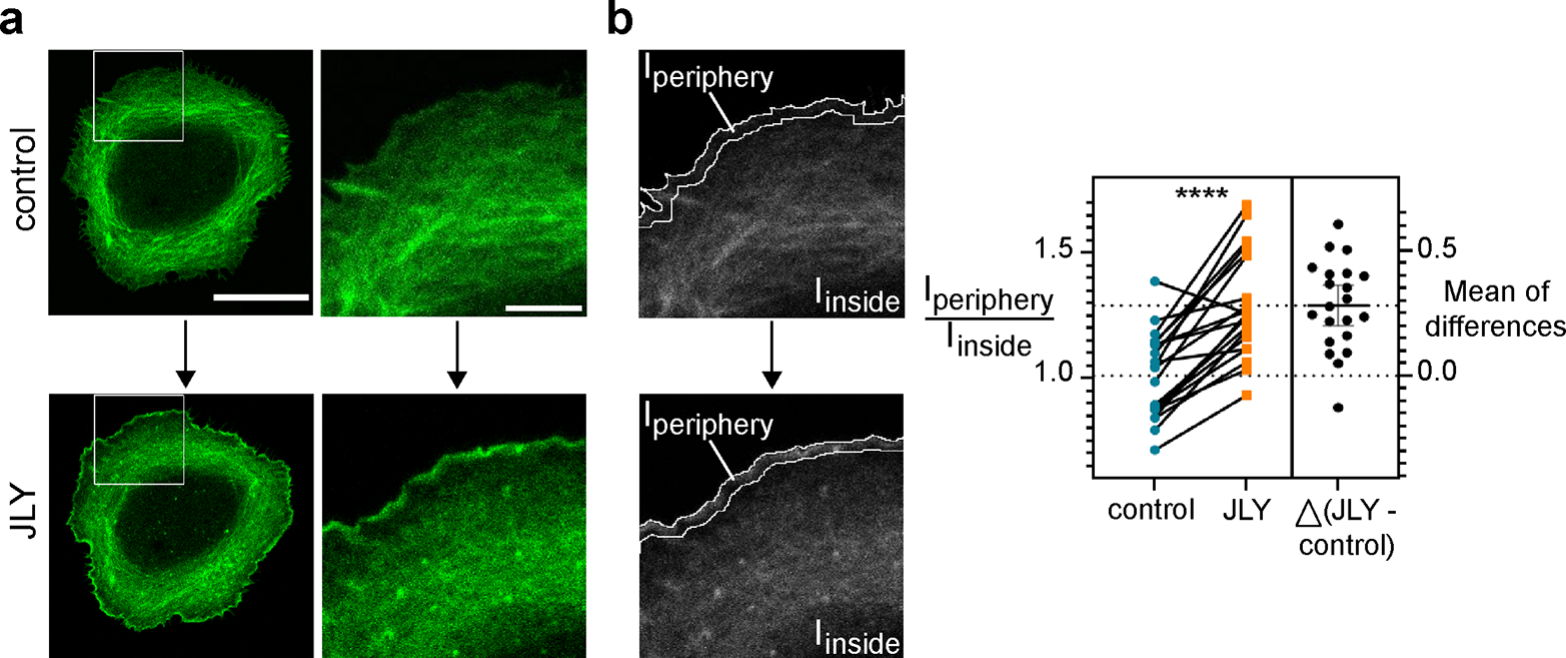
**Relocalization of DARPin 2356_E5 upon cytoskeleton arrest. (a)** Confocal microscopy images of live U2OS expressing mEGFP-DARPin 2356_E5 before and after addition of a pharmacological cocktail that induces rapid actin cytoskeleton arrest. The DARPin accumulated primarily in stress fibers under normal culture conditions; upon the arrest of actin dynamics using the JLY pharmacological cocktail, the DARPin relocalized rapidly to the lamellipodia at the cell periphery. Scale bars: 20 μm; for details scale bars: 5 μm. **(b)** The ratio of fluorescence intensity at the cell periphery (1–2 μm from the cell edge) compared to the cell interior was calculated for cells before and after addition of the JLY pharmacological cocktail. The ratio increased significantly after actin cytoskeleton arrest (One-tailed, paired *t* test: *p* = 0.0001 (****), *n* = 21 cells, *N* = 2 independent experiments).

The above results revealed a correlation between the localization of actin-binding probes and their binding kinetics: probes with faster turnover labeled efficiently lamellipodia and other dynamic structures such as filopodia and podosomes (Movie S4), while probes with long τ_1/2_ were mainly localized in actin stress fibers. This correlation, previously also recognized by others,^19,21^ suggested that the movements of the actin cytoskeleton largely determine the localization of the actin-binding DARPins, and actin probes in general. In order to test this hypothesis, we performed two experiments. First, the actin cytoskeleton was fixed using PFA and cells were then stained with SiR-actin, which was absent from lamellipodia during live cell imaging (Figure 2). In fixed U2OS cells, the dye accumulated efficiently at lamellipodia, in contrast to its distribution toward mature stress fibers in live cells (Figure S15). Second, we added a drug cocktail that rapidly arrested movements of the actin cytoskeleton in live cells^42^ and tracked changes in localization of DARPin 2356_E5, which was absent from lamellipodia at steady state in absence of the drugs. Actin arrest was confirmed via time-lapse imaging: while intracellular vesicle motion was evident, actin-dependent movements, such as filopodia assembly, cell edge protrusions, and membrane ruffling were abolished (Movie S5). Drug treatment led to a redistribution of DARPin 2356_E5 to the cell edge within seconds following actin immobilization (Figure 6a). Accordingly, the ratio of fluorescence intensity of the cell edge to its interior showed a significant increase upon actin arrest (Figure 6b). These results suggest that the localization of actin-binding DARPins is dependent on intracellular actin dynamics.

### DARPins Stain Highly Dynamic Actin Structures

**Figure 7:**
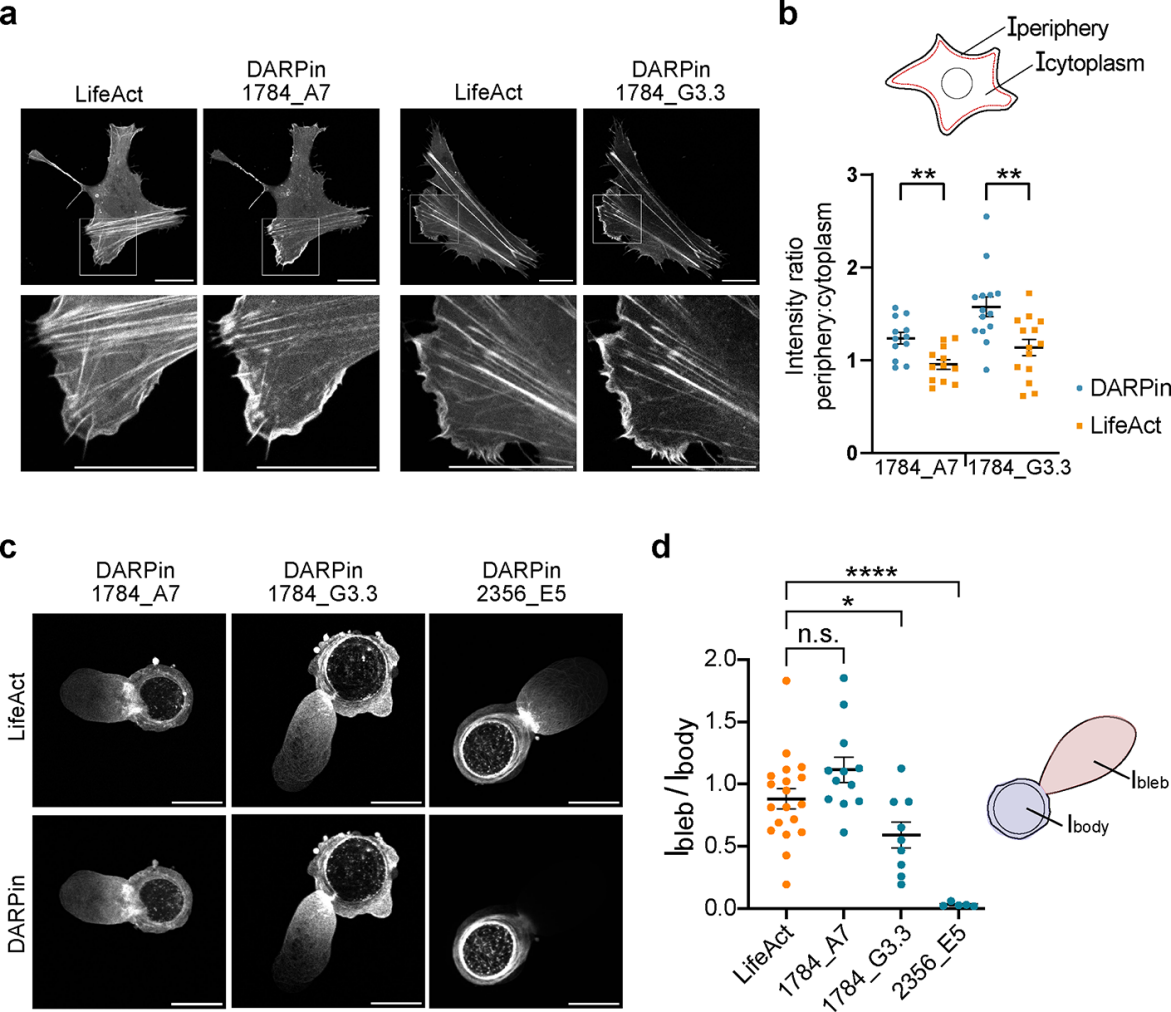
**DARPin labeling of dynamic actin-based cell protrusions. (a)** Confocal microscopy images of U2OS cells transiently cotransfected with mCherry-LifeAct and mEGFP-DARPin 1784_A7 or DARPin 1784_G3.3. The fluorescence intensity of both fluorophores was normalized to 0.35% saturated pixels and the same background intensity. Scale bar: 20 μm. **(b)** The intensity ratio of the cell periphery and interior calculated for DARPins and LifeAct after subtracting the background signal of the respective label. Mean and SEM for DARPin 1784_A7 and LifeAct *n* = 11 cells, *N* = 2 independent experiments, DARPin 1784_G3.3 and LifeAct *n* = 10 cells, *N* = 2 independent experiments. Differences between the intensity ratios from cotransfected cells were determined with Welch’s *t* test, two-tailed: DARPin 1784_A7 and LifeAct *p* = 0.0026 (**) and DARPin 1784_G3.3 and LifeAct *p* = 0.0043 (**). **(c)** Blebbing U2OS cells coexpressing mCherry-LifeAct and a mEGFP-DARPin imaged with spinning disk confocal microscopy. The images were normalized with 0.35% saturated pixels. Scale bar: 20 μm. **(d)** Intensity ratio between the bleb and cell body calculated for DARPin 1784_A7 *n* = 12 cells, *N* = 4 independent experiments, DARPin 1784_G3.3 *n* = 9 cells, *N* = 2 experiment, DARPin 2356_E5 *n* = 5 cells, *N* = 2 independent experiments, and LifeAct *n* = 13, *N* = 5 independent experiments. Mean and SEM are shown. Differences between the intensity ratios of DARPins and LifeAct were determined with an unpaired *t* test. For DARPin 1784_A7 *p* = 0.0819 (n.s.), for DARPin 1784_G3.3 *p* = 0.0441 (*), for DARPin 2356_E5 *p* < 0.0001 (****).

A limitation of existing actin labels is their poor staining of highly dynamic structures at the cell edge, such as filopodia and lamellipodia. Actin is polymerized at the plasma membrane and rapidly flows toward the cell body at speeds typically between 10 and 1000 nm/s.^43^ We evaluated the efficacy of DARPins 1784_A7 and 1784_G3.3 to label cell protrusions in U2OS cells and compared them to LifeAct by calculating the ratio of fluorescence signal at the cell edges, where the cell is protruding, to the cell interior. DARPins 1784_A7 and 1784_G3.3 exhibited higher ratios compared to LifeAct (Figure 7a,b), indicating that they were more efficient in labeling cortical actin. However, DARPin 1784_A7 was not as efficient as LifeAct in labeling stress fibers, in contrast to DARPin 1784_G3.3, which showed similar labeling to LifeAct but improved signal-to-background ratio at the cell edge (Figure 7a). Next, we focused on filopodia and analyzed the fluorescence intensity of the different labels along the length of single filopodia. When fluorescence intensity was normalized to the base of filopodia, all 3 examined labels showed a similar decrease in intensity toward the filopodium tip (Figure S16).

Finally, we examined the capacity of DARPins to label the actin cytoskeleton in blebbing cells. When cells are confined between nonadhesive, flat surfaces, they may switch their morphology and migratory phenotype, often forming large blebs that exhibit very fast retrograde actin flows.^44^ Indeed, confinement of U2OS cells between two glass surfaces coated with poly-l-lysine-*co*-poly(ethylene glycol) (PLL-PEG) and separated by using 4.5 μm beads led to a fraction of cells forming a large bleb (Figure 7c and Movie S6). DARPins 1784_A7 and 1784_G3.3 accumulated in these blebs, highlighting their actin structure and fast retrograde flow; in contrast, DARPin-2356_E5 did not efficiently label actin in blebs, consistent with the results on nonconfined cells. Accordingly, the ratio of fluorescence within the blebs to the fluorescence intensity in the cell body was high for the former DARPins compared to the latter (Figure 7d). Compared to LifeAct, the ability of DARPin 1784_G3.3 to label the actin cortex at the bleb front was lower, while DARPin 1784_A7 showed a similar or slightly higher labeling efficiency of the leading edge compared to LifeAct.

## Discussion

In this study, we generated actin-binding DARPins and demonstrated their application as versatile F-actin labels in living cells. Instead of modifying known actin-binding domains, or modifying small molecular compounds, which is how most actin labels have been identified to date, we screened here a large library for actin-binding candidates based on structure without *a priori* protein design. A recent study reported a similar strategy for the identification of actin-binding proteins, utilizing a library based on the phytocystatin consensus sequence (affimers).^45^ An advantage of such screening approaches is the generation of multiple actin binders with varying affinities, epitopes, and intracellular labeling preferences, yet using binders of similar physicochemical properties, such as size, stability, and structure.

Here, a total of 22 actin-binding DARPins were identified and biochemically validated *in vitro*. Out of these, we ended up with 5 DARPins that exhibited a good signal-to-background ratio in living cells (DARPin 1784_A7, 1784_G3.3, 2356_E5, 2356_F1, and 2358_A11), along with 6 additional DARPins that labeled F-actin only at FAs with high background intracellular fluorescence (DARPin 2359_G12, 2358_B8, 2357_H10, 2357_B2, 2356_A2, and 2358_D10). It is not clear at present why the remaining DARPins from the *in vitro* screen failed to stain any F-actin structures in living cells. Potential reasons explaining the discrepancy between *in vitro* and *in cellulo* binding include competition with other intracellular actin-binding proteins not present in the *in vitro* experiments, low affinity or unsuitable binding kinetics, and alterations in the binding epitopes on actin in the cellular environment compared to the *in vitro* selection. In the case of our second screen against G-actin, an additional reason could be the inaccessibility of the binding site upon polymerization to F-actin. While knowledge of the association/dissociation kinetics and affinities of DARPins on purified actin filaments *in vitro* could provide valuable information to explain our findings, we were unfortunately unable to obtain reproducible results using surface plasmon resonance, despite substantial efforts, presumably due to the highly dynamic nature of immobilized actin filaments.

Our ongoing work aims at identifying the actin binding site of DARPins; knowledge of the precise location at which the different DARPins attach to actin would help us understand the differences in their labeling efficiencies and furthermore predict potential competition with endogenous actin-binding proteins. Even though our initial screen was performed with highly pure actin (99% purity according to the manufacturer), we cannot unambiguously exclude the possibility that DARPins might interact, or synergize, with other ABPs. Of note, during our *in vitro* screens, DARPins demonstrated differential binding to actin depending on the assay used, indicating that the mode of ligand presentation is critical. Among the *in vitro* assays performed, the direct visualization of fluorescent DARPins on F-actin filaments (Figure S6) and the in-solution HTRF assay (Figure S2b) were the best predictor for efficient intracellular labeling.

The intracellular labeling patterns of actin-binding DARPins correlated with their actin-binding dynamics, as these were measured by FRAP, providing a handle to target different actin structures within living cells. While competition with endogenous actin-binding proteins and changes in actin filament conformation might bias the distribution of the DARPins, we propose that the main mechanism is linked to binding kinetics. Filamentous actin inside cells is in a constant flux, polymerizing at the cell edge and rapidly being translocated toward the lamella region, where it depolymerizes or assembles into bundles forming stress fibers. G-actin is also rapidly transported from the lamella back to the cell edge.^46^ This incessant movement affects the positioning of any actin-binding protein when it is in its bound state, as was shown here with DARPins and the widely used actin labels LifeAct and SiR-actin. For example, due to the actin retrograde flow at the cell edge, a probe that binds actin there can move with the same speed toward the cell interior and therefore its distribution will be biased as a function of its binding kinetics.^19,21^ When the on- and off-rates of the probe are high, the probe will have time to detach from the rearward-moving actin and diffuse to label actin at the cell edge; when the rates are low, it will stay stuck to the actin and will be transported with it, resulting in a lack of labeling at the edge. In contrast, actin present in stress fibers exhibits slower translocation relative to the binding kinetics of the probe, and hence, probes with high affinities can also efficiently label these structures. Upon the rapid arrest of the cytoskeleton with a drug cocktail, we observed that a DARPin that primarily accumulated in stress fibers rapidly rearranged and was able to label lamellipodia. Furthermore, SiR-actin showed a completely altered distribution when added to fixed cells compared to living cells, labeling lamellipodia additionally to stress fibers, since all actin structures are immobilized upon fixation. These results consolidate previous findings to the effect that the biased distribution of actin probes is a function of their binding kinetics to actin and the inherent dynamics of the actin cytoskeleton. This general mechanism could also explain previous observations with proposed actin labels^45,47^ and should be kept in mind during data interpretation.

As a consequence of the inherent actin flows, labeling of very dynamic actin structures remains a challenge; some DARPin analogs presented here merit consideration as improved labels for such applications, as exemplified here by the superior performance of DARPin 1784_G3.3 compared to LifeAct for staining cell edge protrusions, including filopodia and lamellipodia. The size of DARPins is significantly larger than that of the LifeAct peptide, suggesting that DARPin diffusion is not a limiting factor for actin labeling in actin-dense regions.

This work complements previous studies in highlighting the potential of DARPins for intracellular applications.^24,26,27,48^ Their small size and stable structure favor their incorporation in fusion constructs, a promising strategy to design cell permeable DARPins using cell-penetrating peptide motifs^49^ or DARPin-based, modular, synthetic actin cross-linkers.^28,50^ The latter approach would benefit from (i) the lack of unwanted interactions encoded in endogenous cross-linkers and (ii) the potential control over binding kinetics enabled by the set of presented DARPins. Overall, the presented actin-binding DARPins constitute a promising tool for use as direct actin labels or as potential components of engineered actin-modulating proteins.

## Conclusions

In summary, we identified actin-binding DARPins through ribosome display and validated their binding using biochemical assays of purified proteins. A subset of these DARPins labeled actin structures when mEGFP-fusions were expressed in living cells, albeit with different localization patterns. The distinct accumulation to dynamic actin structures, such as lamellipodia and blebs, was correlated with DARPin binding kinetics, supporting the hypothesis that intracellular localization of actin probes depends on the inherent movement of the actin cytoskeleton. A direct comparison to the widely used LifeAct probe highlighted the enhanced labeling efficiency and a reduced signal-to-background ratio of one DARPin (1784_G3.3). Overall, DARPins are a valuable addition to our research toolbox for labeling actin and a potential basis for construction of modular actin-regulating tools.

## Materials and Methods

### Materials

A list of reagents and antibodies used in this study is provided in Tables S3 and S4.

### F-Actin Generation

Unlabeled, biotinylated, and rhodamine-labeled G-actin purified from rabbit skeletal muscle as described by Pardee and Spudich^51^ was purchased from Cytoskeleton Inc. (#AKL99 (unlabeled); #AB07 (biotinylated); #AR05 (rhodamine-labeled)). The protein purity was determined by the supplier as >99% by scanning densitometry of Coomassie Blue stained protein on a 12% polyacrylamide gel. Labeling was performed by the covalent linkage of an activated ester of biotin or rhodamine to random surface lysine residues with a determined labeling stoichiometry of approximately 1 biotin and 1 to 2 rhodamine molecules per actin monomer by the supplier.

G-actin was diluted to 0.4 mg/mL in actin buffer (5 mM Tris-HCl pH 8.0, 0.2 mM CaCl_2_, 0.2 mM ATP, 0.5 mM DTT) and depolymerized on ice for 1 h. The polymerization was initiated by the addition of a 1:10 polymerization solution (500 mM KCl, 500 mM MgCl_2_, 10 mM ATP). For biotin-labeled F-actin, 10% of biotinylated G-actin was included. Following an incubation of 1 h at 37 °C, the resulting F-actin was used in subsequent applications.

### DARPin Selection

To generate DARPin binders for actin, biotinylated F-actin (selection 1; DARPin identifier 1781-84_xx) or G-actin (selection 2; DARPin identifier 2356-59_xx) was immobilized alternatingly on either MyOne T1 streptavidin-coated beads (Thermo Scientific) or Sera-Mag neutravidin-coated beads (Cytiva) depending on the selection round. Ribosome display selections were performed as previously described,^31^ using a semiautomatic KingFisher Flex MTP96 well platform. To ensure F-actin stability, the selection and wash buffers were supplemented with 1 mM ATP, 5 mM DTT and in the case of the selection buffer additionally 50 mM MgCl_2_.

The fully synthetic library includes N3C-DARPins with three randomized internal repeats with the original randomization strategy as reported,^37^ but including a stabilized C-cap.^52−54^ Additionally, the library is a 1:1 mixture of DARPins with randomized and nonrandomized N- and C-caps, respectively,^23,55^ and successively enriched pools were ligated in a ribosome display-specific vector.^55^

Selections were performed over four rounds with decreasing concentrations of biotinylated actin and increasing washing steps for the first three cycles, an off-rate selection for high affinity binders using nonbiotinylated target protein in the third cycle, followed by a fourth recovery round with less stringent conditions.^31,56^

### Molecular Cloning

To screen individual DARPins for their binding properties, they were cloned into a prokaryotic expression plasmid and expressed in *E. coli.* For this purpose, the selected pool of DARPins from ribosome display was subcloned by restriction digest with BamHI and HindIII into the pQE30-derived (Qiagen) bacterial expression vectors pQIq-MRGS-8His-SacB-FLAG (selection marker ampicillin) or pQIq_K_MRmyc-TEV-Gly5-SacB-TEV-GGGS-LPETGG-6His (HT-BSF Zurich, Plückthun) replacing the SacB cassette and containing lacIq for expression control. The pQI-MRGS-8His-SacB-FLAG vector was designed to create DARPins with an N-terminal MRGS(H)_8_-tag and a C-terminal FLAG-tag. The pQIq_K_MRmyc-TEV-Gly5-SacB-TEV-GGGS-LPETGG-6His vector was used for the creation of DARPins with an N-terminal myc-tag and a C-terminal sortase recognition sequence combined with a His_6_-tag separated from the DARPin by a flexible G_3_S-TEV-G_3_S linker.

For mammalian expression, the plasmid mEGFP-N1 (a gift from Michael Davidson, Addgene plasmid # 54767) was modified by a NEB Builder high-fidelity (HiFi) DNA assembly in order to add the restriction sites BamHI and HindIII for DARPin insertion to the multiple cloning site, an N-terminal Kozak sequence for mammalian expression and a (G_4_S)_2_-linker between the DARPin insertion site and the C-terminal mEGFP. Thereby, the PCR primers 5′-GTGAGCAAGGGCGAGGAGCTGTTC-3′ and 5′-CTCGAGATCTGAGTCCGGTAGCG-CTAG-5′ were used for plasmid linearization and the additional features were inserted with the dsDNA insert 5′-CTACCGGACTCAGATCTCGGCCACCATGGGATCCGACCTGA-AGCTTAATGGTGGCGGTGGCTCTGGCGGTGGTGGCAGCGTGAGCAAGGGCGAGGAGCTG-3′.

Finally, DARPin 1784_A7 was inserted into the modified vector mEGFP-N1mod using the generated restriction sites BamHI and HindIII.

### DARPin Expression and Purification

*E. coli* XL1-Blue (Stratagene) or *E. coli* DH5α (Thermo Scientific) cells were transformed with individual expression plasmids pQIq-MRGS-8His-SacB-FLAG and pQIq_K_MRmyc-TEV-Gly5-SacB-TEV-GGGS-LPETGG-6His, respectively. Single clones were cultured in TB medium containing 1% glucose and either 100 μg/mL Amp or 50 μg/mL Kan. *lac* operator-controlled protein expression was induced at an OD of 0.6–0.8 with 1 mM IPTG for 5 h at 37 °C. Subsequently, cells were harvested at 3200*g* and lysed for 40 min in IMAC lysis buffer (50 mM sodium phosphate pH 7.4, 300 mM NaCl, 10 mM imidazole, 5% (v/v) glycerol, 1× CelLytic B (Sigma-Aldrich), 0.4 mg/mL lysozyme (Sigma-Aldrich), 80 U/mL Pierce universal nuclease (Thermo Scientific) for IMAC purification. For screening of crude extracts by HTRF or ELISA, the cell pellet was lysed in crude extract lysis buffer (selection 1:250 mM Tris-HCl pH 8.0, 250 mM NaCl, 50 mM MgCl_2_, 50 mg/mL lysozyme, 100 mg/mL *n*-octyl β-d-thioglucopyranoside, 100 U/mL Pierce universal nuclease; Selection 2: B-Per Direct detergent supplemented with 100 U/mL Pierce universal nuclease and 50 mg/mL lysozyme). Resulting crude extracts were cleared by centrifugation and were analyzed by ELISA (selection 1 and 2) and HTRF (selection 1), and after clearance at 3200*g*, lysates (IMAC lysate buffer) were submitted to immobilized metal ion affinity chromatography (IMAC) (selection 1 and 2). The IMAC purification was performed for His-/FLAG-tagged DARPins via HisPur Cobalt Spin plates (Thermo Scientific) and for DAPRin 1784_A7 containing a sortase recognition sequence via a HisTrap HP IMAC column (Cytivia) with coordinated Co^2+^ ions. Following resin equilibration with IMAC buffer (50 mM sodium phosphate pH 7.4, 300 mM NaCl, 10 mM imidazole, 5% (v/v) glycerol), cleared lysates were applied to the IMAC resin and bound protein was washed with IMAC buffer supplemented with additional 100 mM NaCl and 10 mM imidazole. Finally, DARPins were eluted with IMAC buffer supplemented with an additional 490 mM imidazole.

Buffer exchange of purified DARPins to storage buffer (for *in vitro* assays: 10 mM HEPES pH 7.4, 300 mM NaCl, 5% (v/v) glycerol (no glycerol for F-actin binding assay), for cell experiments: 50 mM Tris-HCl pH 7.4, 150 mM NaCl, 5% (v/v) glycerol) was performed with Zeba spin desalting plates or columns (7k MWCO) (Thermo Scientific) according to the manufacturer’s guidelines. Protein concentrations were measured with UV–vis spectroscopy, and purity was verified by SDS-PAGE.

### DARPin Labeling

Purified DARPins were labeled with Atto488 using a sortase A catalytic reaction. The sortase A pentamutant (eSrtA) in plasmid pET29 was a gift from David Liu (Addgene plasmid #75144) and was purified after expression in *E. coli* for 5 h at 30 °C by IMAC (HisTrap HP column (GE Healthcare, 17–5248–01 as described in^57^ using a modified lysis buffer (50 mM Tris pH 7.5, 300 mM NaCl supplemented with 10% (v/v) glycerol, 10 mM imidazole, 5 mM MgCl_2_, 2 U/ml DNaseI (Thermo Scientific), 1 mg/mL lysozyme (Sigma-Aldrich), 10 μg/mL aprotinin (Carl Roth), 10 μg/mL leupeptin (Carl Roth) and 1 mM PMSF (Sigma-Aldrich)). DARPins expressed with a C-terminal LPETGG-His_6_ sortase-recognition sequence (50 μM) were mixed with sortase (2.5 μM) and the labeling peptide G_5_C-Atto488 or G_5_C-Cy5 (250 μM) (PSL peptide specialty laboratories GmbH, ID #2358–12–20) in sortase labeling buffer (50 mM Tris-HCl buffer (pH 7.4), 10 mM CaCl_2_, 150 mM NaCl). The reaction was allowed to proceed at 37 °C under mild shaking. Buffer was exchanged to a phosphate buffer (48 mM K_2_HPO_4_, 4.5 mM KH_2_PO_4_, 14 mM NaH_2_PO_4_; pH 7.2). Separation of DARPins from unreacted peptide was performed with ZebaSpin Desalting columns with a molecular weight cutoff at 7 kDa (Thermo Fisher, 78606). Unlabeled DARPins and sortase were then captured by incubation with magnetic NiNTA beads (Serva) for 15 min at room temperature and removed by magnetic bead separation. The absorbance of the resulting DARPin solution was measured on a NanoPhotometer (Implen, NP80) at the wavelengths of 280, 500, and 650 nm. The concentration of DARPin and the fluorophores Atto488 or Cy5 was calculated with
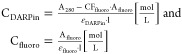
Here, A_280_ is the absorbance value at 280 nm, A_fluoro_ is the absorbance value at the absorbance maximum of the fluorophore that was used in this reaction (either at 500 or 650 nm), CF_fluoro_ is the fluorophore-specific correction factor for absorbance at 280 nm, l is the path length and ε is the molar extinction coefficient. Assuming that no unreacted labeling peptides remained after purification, the labeling efficiency was calculated as the ratio of labeling peptide and DARPin.

The purity of labeled DARPins was verified using SDS-PAGE on a 4–12% Bis-Tris polyacrylamide gel in MES buffer. Following electrophoresis, fluorescence images were recorded at 460, 520, and 630 nm, and a colorimetric image of the Coomassie stained gel was acquired using an Amersham Imager 600 (GE Healthcare Bio-Lifesciences, Uppsala, Sweden).

### Homogeneous time-resolved fluorescence (HTRF)

Crude extracts (1:1000 diluted) from His_8_-DARPin-FLAG-expressing bacteria were incubated with 8 nM biotinylated F-actin, the HTRF donor Streptavidin-Tb cryptate (610SATLB, Cisbio) and its acceptor mAb anti-FLAG M2-d2 (61FG2DLB, Cisbio) for 30 min or 2 h at room temperature in Taglite assay buffer (Cisbio), supplemented with 50 mM MgCl_2_, 1 mM ATP and 5 mM DTT for F-actin stability. FRET signals were measured at 620 and 665 nm with a Varioskan LUX Multimode Microplate Reader (Thermo Scientific) using a delay time of 60 μs, an integration time of 200 μs, and a measurement time of 1000 ms. The final 665/620 HTRF ratio was obtained by dividing the acceptor signal (665 nm) by the donor signal (620 nm) and multiplying the resulting value by 10,000. Data were obtained from a single run for each DARPin.

### Enzyme-linked immunosorbent assay (ELISA)

Crude extracts of 380 DARPin clones or selected IMAC-purified DARPins (His_8_-DARPin-FLAG) were screened for their ability to bind to actin by an enzyme-linked immunosorbent assay (ELISA). For this purpose, crude extracts of DARPin clones or purified DARPins diluted (1:1000 (screens 1 and 2) or 100 nM (quantitative ELISA)) in PBS-TB (PBS pH 7.4, 0.1% (v/v) Tween-20, 0.2% (w/v) bovine serum albumin (BSA) (supplemented with 1 mM ATP, 5 mM DTT and 50 mM MgCl_2_ (for ELISA with F-actin)/2 mM CaCl_2_ (for ELISA with G-actin) for actin stability) were incubated with 50 nM biotinylated F-actin (screen 1) or 50 nM G-actin (screen 2 and quantitative ELISA) immobilized on precoated neutravidin (screen 1 and 2) or streptavidin (quantitative ELISA) for 1 h at room temperature. Subsequently, target-specific binding of DARPins was detected after each 1 h incubation at room temperature with a mouse α-FLAG RGS(His)_4_ IgG_1_ (clone M2) (Sigma-Aldrich; F3165) (1:5000 in PBS-TB) and an alkaline phosphatase -conjugated polyclonal goat α-mouse-IgG antibody as secondary antibody (Sigma-Aldrich; A3562) (1:10000 in PBS-TB). Target-specific binding of DARPins was analyzed by following the color change of the hydrolysis of 3 mM para-nitrophenyl phosphate (pNPP) substrate in pNPP buffer (50 mM NaHCO_3_, 50 mM MgCl_2_·(H_2_O)_6_) using a wellplate reader (Tecan). Color change was determined by the difference in the optical density at 405 and 540 nm. Clones with minimum signals 3-fold over background were considered as positive. Data result from a single run of each DARPin.

### Analytical size exclusion chromatography (SEC)

Size exclusion chromatography of 50 μL of His_8_-DARPin-FLAG proteins (10 μM) was performed on a LC1200 HPLC system (Agilent) using a Superdex 200 Increase 5/150 GL column (GE Healthcare) and a flow rate of 0.4 mL/min with PBS, 400 mM NaCl as running buffer. The absorbance at 280 nm was recorded. Chromatograms were produced from single runs for each DARPin.

### *In vitro* F-actin polymerization assay

Pyrene-labeled G-actin purified from rabbit skeletal muscle was purchased from Hypermol. The protein purity was determined by the supplier with >99% by scanning densitometry. Labeling was performed by the covalent linkage of a fluorescent pyrenyl group to Cys374 at the C-term of the actin molecule with a labeling stoichiometry of approximately 10% as determined by the supplier. For the polymerization assay, 1 mg/mL of pyrene-labeled G-actin was depolymerized on ice for 20 min and dialyzed (10k MWCO) overnight at 4 °C against 100 sample volumes of actin assay buffer (2 mM Tris pH 8.2, 0.1 mM KCl, 0.2 mM DTT, 0.4 mM ATP). Next, the solution was cleared by centrifugation (15,000 *g* for 30 min at 4 °C), and the supernatant containing nonpolymerized pyrene-actin was diluted in actin assay buffer. Polymerization was initiated by the addition of a mixture of His_8_-DARPin-FLAG proteins in 10 mM HEPES pH 7.4, 300 mM NaCl, 5% (v/v) glycerol and assay polymerization solution (1 M KCl, 20 mM MgCl_2_, 100 mM imidazole). The volume ratio of G-actin, DARPin and assay polymerization solution was 7.93/1.07/1, and final concentrations of G-actin and DARPins were 7.5 and 2.5 μM, respectively. As a positive control the DARPin sample volume was replaced by actin assay buffer, and as a buffer control it was replaced by 10 mM HEPES pH 7.4, 300 mM NaCl, 5% (v/v) glycerol. For the negative control, the sample volume of DARPin and the assay polymerization solution was replaced by actin assay buffer. Polymerization was monitored by an increase in fluorescence due to pyrene stacking (excitation 364 nm/emission 409 nm) every 1 min using a Tecan SparkTM microplate reader (Tecan) at 29 °C. Three independent experiments were performed per DARPin. For the determination of the final polymerization level, fluorescence values 15 min after polymerization initiation were normalized to the positive control of each experiment and an unpaired, parametric students *t* test was performed to compare final polymerization levels using GraphPad Prism version 9.3.1. Significance was defined as *p* ≤ 0.05.

### *In vitro* F-actin staining

Three μM freshly prepared, unlabeled F-actin in actin buffer was incubated for 1 h at 25 °C with 0.3 μM His_8_-DARPin-FLAG followed by a second incubation (1 h at 25 °C) of the actin-DARPin mix with 0.12 μM FITC-labeled anti-His_6_-tag antibody (mouse IgG_1_, clone AD1.1.10) (Thermo Scientific). DARPins were provided in 10 mM HEPES at pH 7.4, 300 mM NaCl; the antibody solution was PBS-based. F-actin, DARPin and antibody were mixed in a volume ratio of 7:1:2. Finally, a drop of the solution mix was placed on a glass coverslip, and subsequently samples were imaged with a Zeiss LSM 900 confocal fluorescence microscope equipped with a 20x air objective Plan-Apochromat 20*x*/0.8 M27 (Zeiss). Three independent experiments were performed; the data presented originate from one representative set. For optimal contrast and brightness, images were processed equally with the software Fiji.

### Cell culture

U2OS cells, a human osteosarcoma cell line, was purchased from the DMSZ-German collection of microorganisms and cell cultures GmbH and a gift from the laboratory of Kai Johnsson (Max Planck Institute for Medical Research, Heidelberg, Germany). U2OS were cultured in McCoy 5A medium supplemented with 10% fetal bovine serum (FBS) and 100 U/ml penicillin-streptomycin at 37 °C and 5% CO_2_.

Immortalized fibroblasts derived from kidneys of 21-days old Fermt1^flox/flox^ Fermt2^flox/flox^ mice were kindly provided by the laboratory of Prof. R. Fässler (Department of Molecular Medicine, Max Planck Institute of Biochemistry, Martinsried, Germany) [Theodosiou et al, Elife 2016]. Fibroblasts were cultured as subconfluent monolayers in Dulbecco’s modified Eagle’s medium (DMEM) supplemented with 10% FBS, 1 mM sodium pyruvate, and 100 U/ml penicillin-streptomycin in a 5% CO_2_ incubator at 37 °C.

Primary human dermal fibroblasts (pHDF) were purchased from the American Type Culture Collection (ATCC). pHDF cells were cultured in DMEM supplemented with 10% FBS and 100 U/ml penicillin-streptomycin at 37 °C and 5% CO_2_.

### Microinjection

Glass-bottom Petri dishes were marked on the bottom side using a diamond pen to help identify injected cells. The glass was coated with 10 μg/mL fibronectin in PBS for 1 h at room temperature. Fibroblasts were seeded in these Petri dishes at a density of 6,500 cells/cm^2^. After overnight incubation, the cells were washed with PBS and CO_2_-independent medium (Gibco, 18045–054) supplemented with 10% FBS was added. The Petri dish was transferred to an inverted Zeiss Axio observer Z1 microscope, on which a microinjection device was mounted.

The injection capillary (FemtoTip) was loaded with 3 μL of Cy5- or Atto-488-labeled DARPins in phosphate buffer (48 mM K_2_HPO_4_, 4.5 mM KH_2_PO_4_, 14 mM NaH_2_PO_4_; pH 7.2) and connected to a pressure-controlled injection device (FemtoJet). Cells were injected with a pressure of 150 hPa and a compensation pressure of 20 hPa for 0.5 or 1 s. Typically, 10–15 cells were injected within 1 h. Successful injections were identified by epifluorescence imaging using a 40x objective (EC Plan-Neofluar NA = 0.75); the location of injected cells was noted and the Petri dish was transferred to and imaged on a Zeiss LSM880 confocal microscope, equipped with a Plan-Apochromat 63*x*/1.4 NA objective and on-stage incubation at 37 °C.

### Cell transfections

U2OS cells (8 × 10^4^ cells) were seeded in 12-well culture plates for transient transfection. Twenty-four h after seeding, the cells were transiently transfected using 800 ng of plasmid DNA mixed with 1.6 μL of Lipofectamine 3000 and 1.6 μL of P3000 reagent (Invitrogen) according to the manufacturer’s protocol. Plasmids used for cell transfection were mCherry-LifeAct (Ibidi #60101 with pCMV promotor), mCherry-Paxillin-22 (a gift from Michael Davidson; Addgene plasmid # 55114)^58^ and the DARPins inserted in the multiple cloning site of the modified mEGFP-N1. Cells were harvested 24 h after lipofection for further use.

### Flow Cytometry

Cells (transfected and controls) were washed once with PBS and detached from 12-well plates by incubating with 150 μL of 0.05% trypsin-EDTA for 2 min at 37 °C. Cells were then mixed with 2 mL of supplemented culture medium, pelleted by centrifugation, and resuspended in 300 μL of 1% (w/v) BSA in PBS. The cell suspension was filtered through a flow cytometry tube filter cap and stored in ice until analysis. Flow cytometry was performed on an LSRFortessa X-20 instrument (BD Biosciences). 1 × 10^4^ events/sample were acquired, and the results were analyzed using the software FlowJo (Version 10.6.1). Transfection efficiency was calculated on the subpopulation of events that were determined as living cells based on their FSC to SSC ratio. For each plasmid, the transfection efficiency was calculated as the percentage of living cells that exceeded the mEGFP levels of the untransfected controls. The mean transfection efficiency with mEGFP-DARPins was calculated from two independent experiments with 6 randomly chosen actin-DARPins.

### Colocalization Studies

U2OS cells expressing mEGFP-DARPins were seeded on fibronectin-coated glass for 3 h and then fixed with 4% paraformaldehyde (PFA) in PBS at room temperature for 20 min. Cells were washed 3 times with PBS, the cell membrane was permeabilized with 0.1% Triton-X-100 for 5 min at room temperature, and samples were incubated with 1% (w/v) BSA in PBS for 1 h at room temperature. Filamentous actin (F-actin) was stained by incubating with 1 μg/mL TRITC-phalloidin (Sigma, #P1951) in PBS for 30 min. Cortactin and paxillin were stained with a 1:150 dilution of anti-cortactin IgG (SantaCruz, SC-11408) or 1:100 dilution of anti-paxillin IgG in 1% (w/v) BSA for 1 h. Cells were then washed three times with PBS, and the secondary anti-rabbit IgG AlexaFluor568 was added in a 1:150 dilution for 1 h in 1% (w/v) BSA in the dark. Cells were washed three times with PBS and mounted with mowiol on a carrier glass.

Images of stained cells were acquired using a Zeiss LSM 880 confocal microscope with a Plan-Apochromat 63x/1.4 NA objective. The degree of colocalization for DARPins and F-actin was determined by correlating the mEGFP and TRITC intensity in each pixel within a manually annotated cell and calculating the Pearson Correlation Coefficient using the EzColocalization Plugin (Stauffer et al. 2018) for Fiji (Schindelin et al. 2012).

### Actin Dynamics Arrest

Transiently transfected U2OS cells expressing mEGFP-DARPins were seeded on FN-coated, chambered glass-bottom microscopy slides (Nunc Lab-Tek II) for 1–2 h. The medium was exchanged to 200 μL of supplemented CO_2_-independent medium, and the cells were transferred to the Zeiss LSM 880 confocal microscope equipped with a heating stage. Z-stacks of single cells were acquired before drug addition. Then, 25 μL of the small molecule inhibitor Y27632 (100 μM) for 10 min was added, followed by addition of 25 μL of latranculin B (50 μM) and jasplakinolide (80 μM) to give final concentrations of 10 μM, 5 μM, and 8 μM for Y27632, latranculin B, and jasplakinolide, respectively. Immediately after, z-stacks of the same cells were acquired. The fluorescence intensity ratio at the cell edge to the intensity at the cell interior was calculated for each cell using a custom-made ImageJ plugin. Briefly, two regions of interest (ROIs) were defined for each cell—one corresponding to the cell cortex and one for the cell interior (total cell area – cell cortex)—and the intensity was calculated for each ROI.

### Stimulated Emission Depletion (STED) Imaging

U2OS cells transiently transfected with DARPin 1784_A7 or 2356_E5 were seeded on glass coverslips that were coated with 10 μg/mL fibronectin. Cells were fixed with 4% PFA 3 h after seeding and washed with PBS, and quench fixative (100 mM glycine, 100 mM NH_4_Cl in PBS) was added for 5 min. Cells were then washed and blocked with 1% BSA for 30 min and washed again, and the nanobody Fluotag x4 GFP coupled with the dye Star635P was added to the cells in a 1:250 dilution for 1 h in the dark. Cells were washed and mounted in mowiol. The sample was left to dry overnight prior to STED imaging. Imaging was performed on an Abberior Expert Line (Abberior Instruments GmbH, Göttingen, Germany) built on a motorized inverted microscope IX83 (Olympus, Tokyo, Japan). The microscope is equipped with pulsed STED lasers at 595 and 775 nm shaped by Spatial Light Modulators (SLMs), and with 355, 405, 485, 561, and 640 nm excitation lasers. Spectral detection is performed with avalanche photodiodes (APDs). Images were acquired with a 100x/1.40 UPlanSApo Oil immersion objective lens (Olympus). Pixel size was 30 nm for all of the images. Laser powers and dwell times were optimized for each sample.

### Fluorescence Recovery after Photobleaching (FRAP)

U2OS cells expressing mEGFP-DARPins or mCherry-LifeAct were seeded on chambered glass-bottom microscopy slides (Nunc Lab-Tek II), which were coated with 10 μg/mL fibronectin in PBS overnight at 4 °C. FRAP experiments were performed on a Zeiss LSM 880 confocal microscope equipped with a Plan-Apochromat 63x/1.4 NA objective and an incubation chamber set at 37°C and 5 % CO_2_. FRAP measurements were performed on cells 3–6 h after cell seeding as follows: a square 0.53 × 0.53 μm^2^ region of fluorescent proteins on stress fibers or lamellipodia was bleached using a short (540.2 ms) pulse of high-power laser at the wavelength corresponding to the label used (488 nm for mEGFP, 514 and 561 nm for mCherry). Images were acquired before and after the bleaching event at frame rates of 1.0, 3.9, 15.4, or 19.5 frames/s, depending on the DARPin bleached.

FRAP analysis was performed using Fiji, and the resulting data were analyzed and fitted with the web-based tool easyFRAP.^59^ First, the background intensity, which was calculated from a region outside the cell, was subtracted from the bleached region of interest (ROI) and from a control region outside the bleached spot (ctrl). Then, the intensity in the region of interest was corrected for bleaching due to imaging and for different starting intensities:
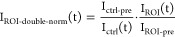
where I_ctrl-pre_ and I_ROI-pre_ are the average intensities before the bleaching event in the control region and the ROI, respectively. The intensity versus time data were fitted with a single-exponential curve to determine the half recovery time (τ_1/2_) and mobile fraction.

### Stable Bleb Cells

Microscope glass slides (76 × 26 mm) and glass coverslips (22 × 22 mm) were coated with 100 μg/mL poly-l-lysine-*co*-poly(ethylene glycol) (PLL-PEG) in PBS for 16 h at 4 °C and were washed three times with PBS. U2OS cells transiently transfected with mEGFP-DARPins or mCherry-LifeAct were diluted to 5·10^5^ cells/ml in CO_2_-independent medium with 10% FBS. 1 μl of a suspension of 4.5 μm sized polystyrene beads was added to 200 μL of cell suspension. Then, 2 μL of cell-bead suspension was added to the PLL-PEG coated microscope slide. Cells were confined by placing the PLL-PEG coated coverslip on the solution. Images of confined cells were acquired immediately using a Nikon Eclipse Ti2-E/Yokogawa CSU-W1 spinning disk confocal microscope with a Nikon CFI-Apochromat TIRF 60X/1.49 NA oil objective and an Okolab Cage Incubator at 37 °C. Images were acquired at exposure times from 50 ms to 200 ms.

The mean fluorescence intensity of stable-bleb cells was measured with Fiji in the cell body and the cell bleb. The mean background fluorescence intensity was subtracted, and the ratio of fluorescence intensity in the stable bleb and cell body was calculated.

### Statistical Analysis

GraphPad Prism (GraphPad Software, San Diego CA) was used to conduct all statistical analyses and create graphs. The applied statistical tests are noted in the figure legends. *p*-values are classified as follows: *p < 0.05; **p < 0.01; ***p < 0.001; ****p < 0.0001, unless exact *p*-values are noted in the figure legends.

## Supporting Information Available

Figures S1–S16: Sequence alignment of actin proteins; ELISAs and HTRF screens; analytic size exclusion chromatography; *in vitro* F-actin polymerization assay; *in vitro* F-actin staining with DARPins; sortase-mediated DARPin labeling; DARPin localization in three mammalian cell lines; FACS of mEGFP-DARPin expressing U2OS cells; localization of 17 DARPins in living U2OS cells; STED imaging of DARPins; calculation of the enrichment ratio of DARPins in lamellipodia; co-injection of two actin-binding DARPins; SiR-actin localization in live and fixed cells; comparison of filopodia labeling with DARPins and LifeAct in live cells. Tables S1–S4: Amino acid sequence of 17 DARPins; DARPin-dependent labeling of actin structures; reagent list; antibody list. (PDF)
Movie S1. pHDF cells microinjected with DARPin 1784_A7 and labeled with SiR-actin. (AVI)
Movie S2. U2OS cells expressing mEGFP-DARPin 1784_A7 on soft silicone elastomers. (AVI)
Movie S3. U2OS cells expressing mEGFP-DARPin 2356_E5 on soft silicone elastomers. (AVI)
Movie S4. Detail of U2OS cells expressing mEGFP-DARPin 1784_A7. (AVI)
Movie S5. U2OS cells expressing mEGFP-DARPin 2356_E5 after actin cytoskeleton arrest with the JLY drug cocktail. (AVI)
Movie S6. Spinning disk confocal microscopy imaging of stable-blebs in U2OS cells expressing mEGFP-DARPins or mCherry LifeAct. (AVI)

## Supplementary Material

nn3c12265_si_001.pdf

nn3c12265_si_002.avi

nn3c12265_si_003.avi

nn3c12265_si_004.avi

nn3c12265_si_005.avi

nn3c12265_si_006.avi

nn3c12265_si_007.avi

## Abbreviations

AAV: adeno associated viral
ABPs: actin-binding proteins
BSA: bovine serum albumin
DARPins: designed ankyrin repeat proteins
DMEM: Dulbecco’s modified Eagle’s medium
ELISA: enzyme-linked immunosorbent assay
eSrtA: sortase A pentamutant
ER: enrichment ratio
F-actin: filamentous actin
FA: focal adhesions
FACS: fluorescence-activated cell sorting
FBS: fetal bovine serum
FRAP: fluorescence recovery after photobleaching
G-actin: globular actin
HTRF: homogeneous time-resolved fluorescence
IMAC: immobilized ion metal affinity chromatography
mEGFP: monomeric enhanced green-fluorescent protein
PBS: phosphate-buffered saline
PCC: Pearson’s correlation coefficient
PFA: paraformaldehyde
pHDF: primary human dermal fibroblasts
PLL-PEG: poly-l-lysin-*co*-poly(ethylene glycol)
pNPP: para-nitrophenyl phosphate
ROI: region of interest
SEC: size exclusion chromatography
STED: stimulated emission depletion
τ_1/2_: half-recovery time
U2OS: human bone osteosarcoma epithelial cells
