# Supplementary material for: Designed Ankyrin Repeat Proteins as Actin Labels of Distinct Cytoskeletal Structures in Living Cells
Source: ACS Nano. 2024 Mar 15;18(12):8919–33. doi: 10.1021/acsnano.3c12265 (PMC10976963; doi:10.1021/acsnano.3c12265)
Supplement: Supplementary file 1 — nn3c12265_si_001.pdf [file nn3c12265_si_001.pdf]

Supporting Information

Designed Ankyrin Repeat Proteins as Actin Labels of Distinct Cytoskeletal Structures in Living Cells

Julia R. Ivanova[1,4,5#], Amelie S. Benk[1#], Jonas V. Schaefer[2,6], Birgit Dreier[2], Leon O. Hermann[1], Andreas Plückthun[2], Dimitris Missirlis[1,3], Joachim P. Spatz[1,3,5]**

# contributed equally

1. Department of Cellular Biophysics, Max Planck Institute for Medical Research; postal address: Jahnstr. 29, D-69120 Heidelberg, Germany

2. Department of Biochemistry, University of Zurich, Winterthurerstr. 190, 8057 Zurich, Switzerland

3. Institute for Molecular Systems Engineering and Advanced Materials, Heidelberg University; postal address: INF 225, D-69120 Heidelberg, Germany

4. Heidelberg University, Faculty of Biosciences, 69120 Heidelberg, Germany

5. Max Planck School Matter to Life, Jahnstrasse 29, 69120 Heidelberg, Germany

6. CSL Behring AG, 3014 Bern, Switzerland

* corresponding authors

Joachim P. Spatz: spatz@mr.mpg.de

Dimitris Missirlis: dimitris.missirlis@mr.mpg.de

```
P68135 | Rabbit skeletal muscle actin
P68133 | Human skeletal muscle actin
P60709 | Human cytoplasmic actin

MCDEDETTALVCDNGSGLVKAGFAGDDAPRAVFPSIVGRPRHQGVMVGMGQKDSYVGDEAQSK
MCDEDETTALVCDNGSGLVKAGFAGDDAPRAVFPSIVGRPRHQGVMVGMGQKDSYVGDEAQSK
--MDDDIAALVVDNGSGMCKAGFAGDDAPRAVFPSIVGRPRHQGVMVGMGQKDSYVGDEAQSK

RGILTLKYPIEHGIITNWDDMEKIWHHTFYNELRVAPEEHPTLLTEAPLNPKANREKMTQIMF
RGILTLKYPIEHGIITNWDDMEKIWHHTFYNELRVAPEEHPTLLTEAPLNPKANREKMTQIMF
RGILTLKYPIEHGIVTNWDDMEKIWHHTFYNELRVAPEEHPVLLTEAPLNPKANREKMTQIMF

ETFNVPAMYVAIQAVLSLYASGRTTGIVLDSGDGVTHNVPIYEGYALPHAIMRLDLAGRDLTD
ETFNVPAMYVAIQAVLSLYASGRTTGIVLDSGDGVTHNVPIYEGYALPHAIMRLDLAGRDLTD
ETFNTPAMYVAIQAVLSLYASGRTTGIVMDSGDGVTHTVPIYEGYALPHAILRLDLAGRDLTD

YLMKILTERGYSFVTTAEREIVRDIKEKLCYVALDFENEMATAASSSSLEKSYELPDGQVITI
YLMKILTERGYSFVTTAEREIVRDIKEKLCYVALDFENEMATAASSSSLEKSYELPDGQVITI
YLMKILTERGYSFTTTAEREIVRDIKEKLCYVALDFEQEMATAASSSSLEKSYELPDGQVITI

GNERFRCPETLFQPSFIGMESAGIHETTYNSIMKCDIDIRKDLYANNVMSGGTTMYPGIADRM
GNERFRCPETLFQPSFIGMESAGIHETTYNSIMKCDIDIRKDLYANNVMSGGTTMYPGIADRM
GNERFRCPEALFQPSFLGMESCGIHETTFNSIMKCDVDIRKDLYANTVLSGGTTMYPGIADRM

QKEITALAPSTMKIKIIAPPERKYSVWIGGSILASLSTFQQMWITKQEYDEAGPSIVHRKCF
QKEITALAPSTMKIKIIAPPERKYSVWIGGSILASLSTFQQMWITKQEYDEAGPSIVHRKCF
QKEITALAPSTMKIKIIAPPERKYSVWIGGSILASLSTFQQMWISKQEYDESGPSIVHRKCF
```

Figure S1. Sequence alignment of actin proteins from different species/tissues. Amino acid sequences of alpha skeletal muscle from human (*Homo sapiens*) and rabbit (*Oryctolagus cuniculus*) origin and cytoplasmic actin from human (*Homo sapiens*) were retrieved from the UniprotKB/Swiss-Prot database (accession numbers P68135, P68133, P60709) and aligned using Clustal Omega (Clustalo Version 1.2.4; https://www.ebi.ac.uk/Tools/msa/clustalo/). The sequence identity is 100 % for human versus rabbit skeletal muscle actin and 93.33 % for human cytoplasmic actin versus rabbit skeletal muscle actin, respectively. Differences in amino acids are highlighted in orange.

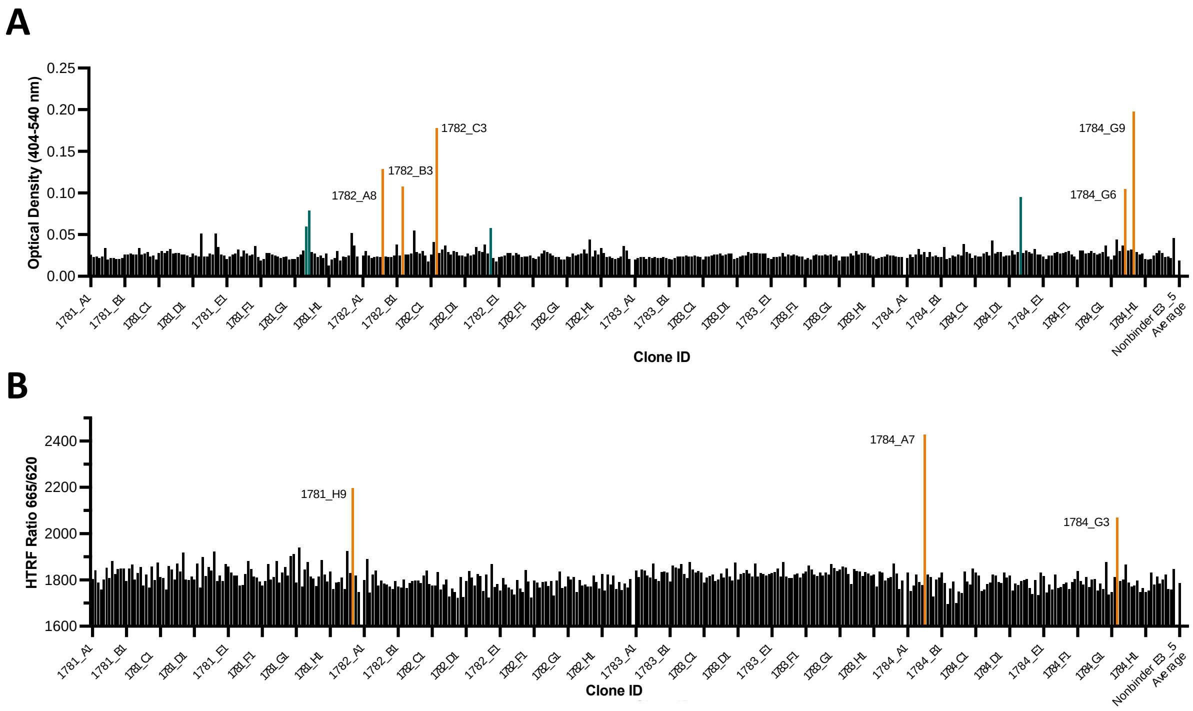

Figure S2. Screening for actin-binding DARPin clones from selection 1 (F-actin) by ELISA and HTRF. Crude extracts of 380 DARPin clones (N-terminal His_8-Tag, C-terminal FLAG tag) from selection 1 were analyzed by ELISA (**A**) and HTRF (**B**) against F-actin for their actin-binding capability (N=1). Clones considered as positive are marked in orange (selected for subsequent sequencing) and green (not sequenced).

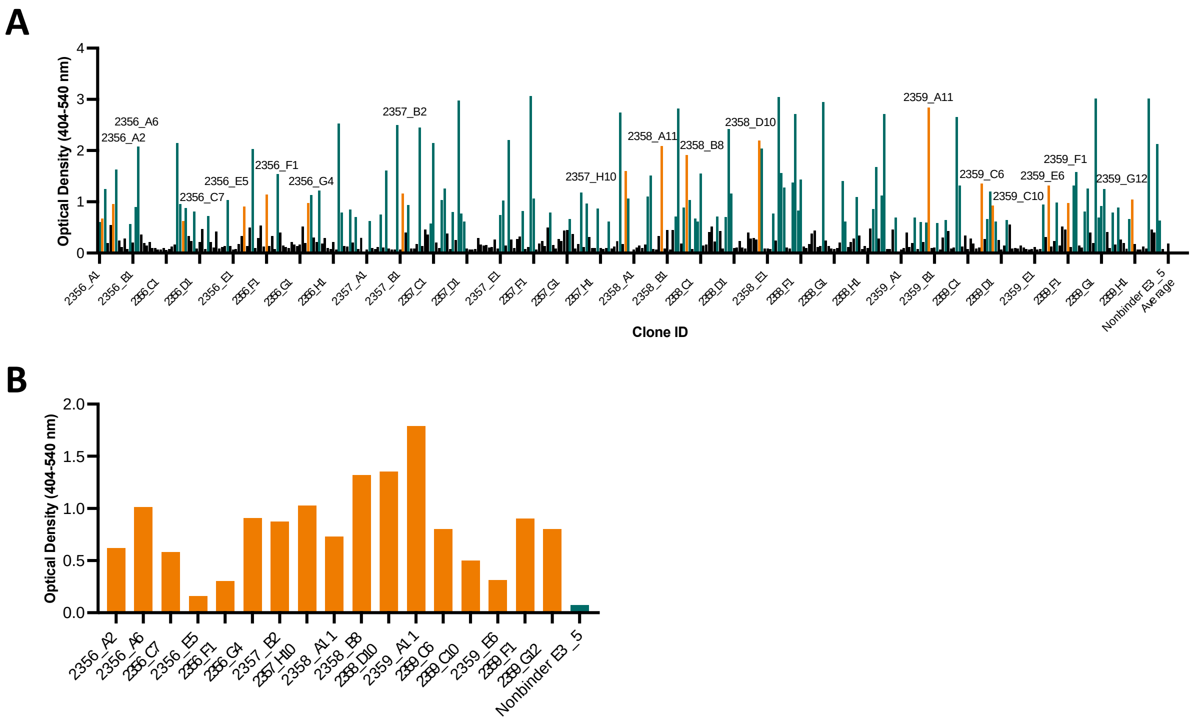

Figure S3. Screening for actin-binding DARPin clones from selection 2 (G-actin) via ELISA. DARPin clones (N-terminal His_8-Tag, C-terminal FLAG tag) were analyzed by ELISA against immobilized G-actin. **A.** Qualitative ELISA of crude extracts from 380 DARPin clones (N=1). Clones with minimum signals threefold over background (marked in green) were considered as positive binders; from these, 32 were randomly selected and subjected to sequencing. Sequenced individual clones are marked in orange. **B.** Quantitative ELISA results for selected, IMAC-purified DARPins (N=1).

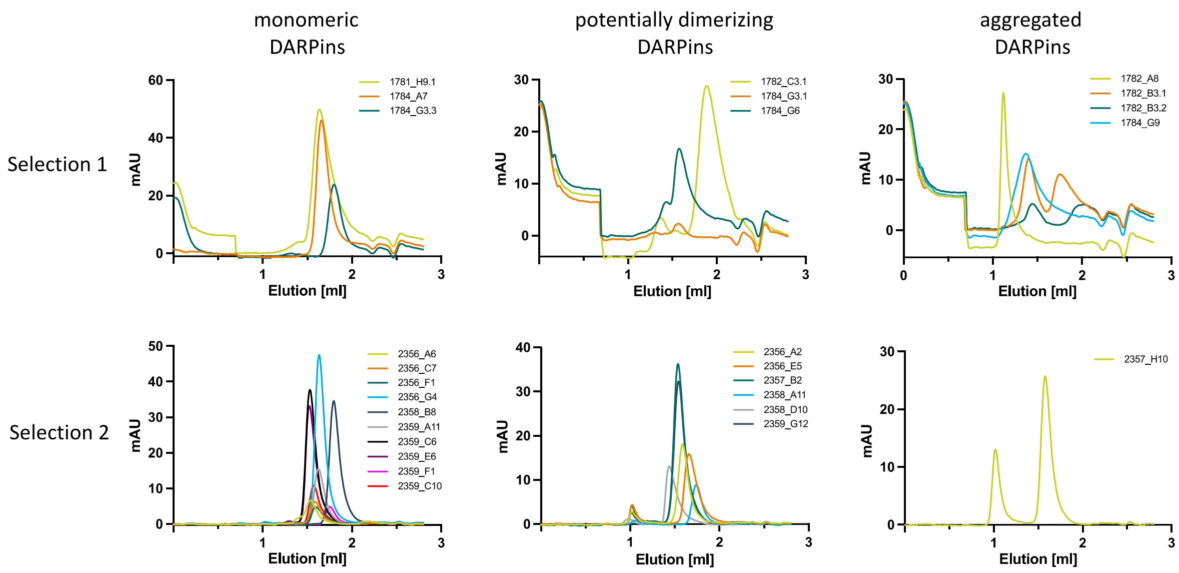

Figure S4. Analytical size exclusion chromatography. Chromatograms of analytical size exclusion chromatography of IMAC-purified His$_8$-DARPin-FLAG proteins on a Superdex 200 Increase 5/150 GL column. The absorption at 280 nm is plotted. Due to different amino acid compositions of the DARPins molar absorption coefficients differ, which effect maximum signal intensities. 13 DARPins show a monomeric behavior, 9 DARPins tend to form dimers and 5 DARPins aggregate, resulting in total in 22 DARPins, which could be characterized further.

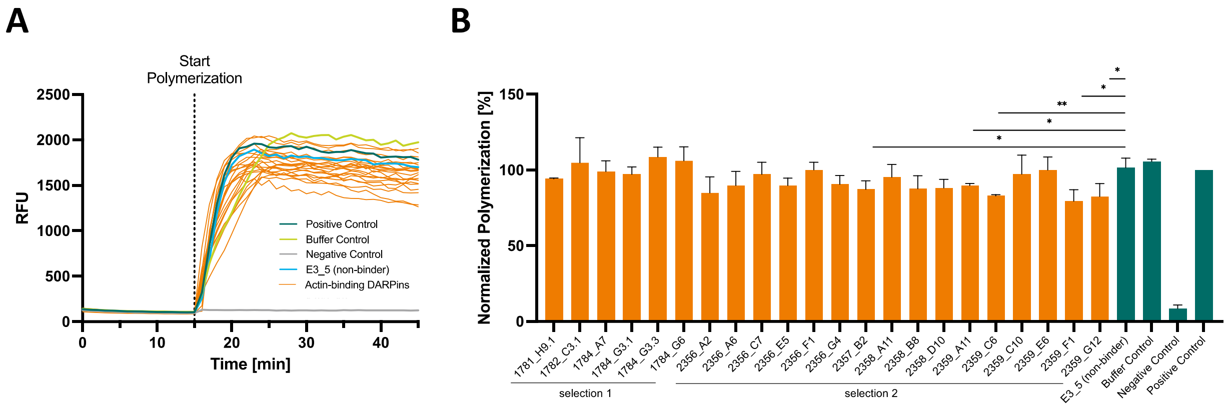

Figure S5. *In vitro* polymerization F-actin assay in the presence of actin-binding DARPins. Polymerization of pyrene-labeled G-actin in the presence of actin-binding DARPins (molar ratio actin:DARPin 3:1) monitored by the increase in pyrene fluorescence in the presence of DARPins or under control conditions. The positive and negative control consist of actin in actin buffer supplemented with or without polymerization solution, respectively. The buffer control contains actin in actin buffer with polymerization solution and DARPin buffer without DARPins. **A.** F-actin polymerization kinetics from one out of three independent experiments are presented. All actin-binding DARPins allow the *in vitro* polymerization of F-actin. **B**. Final polymerization level of pyrene-labeled G-actin 15 min after polymerization initiation and normalized to the positive control for each measurement set. 5 of 22 DARPins showed a noteworthy influence on the maximum *in vitro* polymerization level of F-actin compared to the non-binder DARPin E3_5. N= 3, Error bars: ± SD; parametric, unpaired students t-test p-values: < 0.01: **; < 0.05: *; >0.05: not significant.

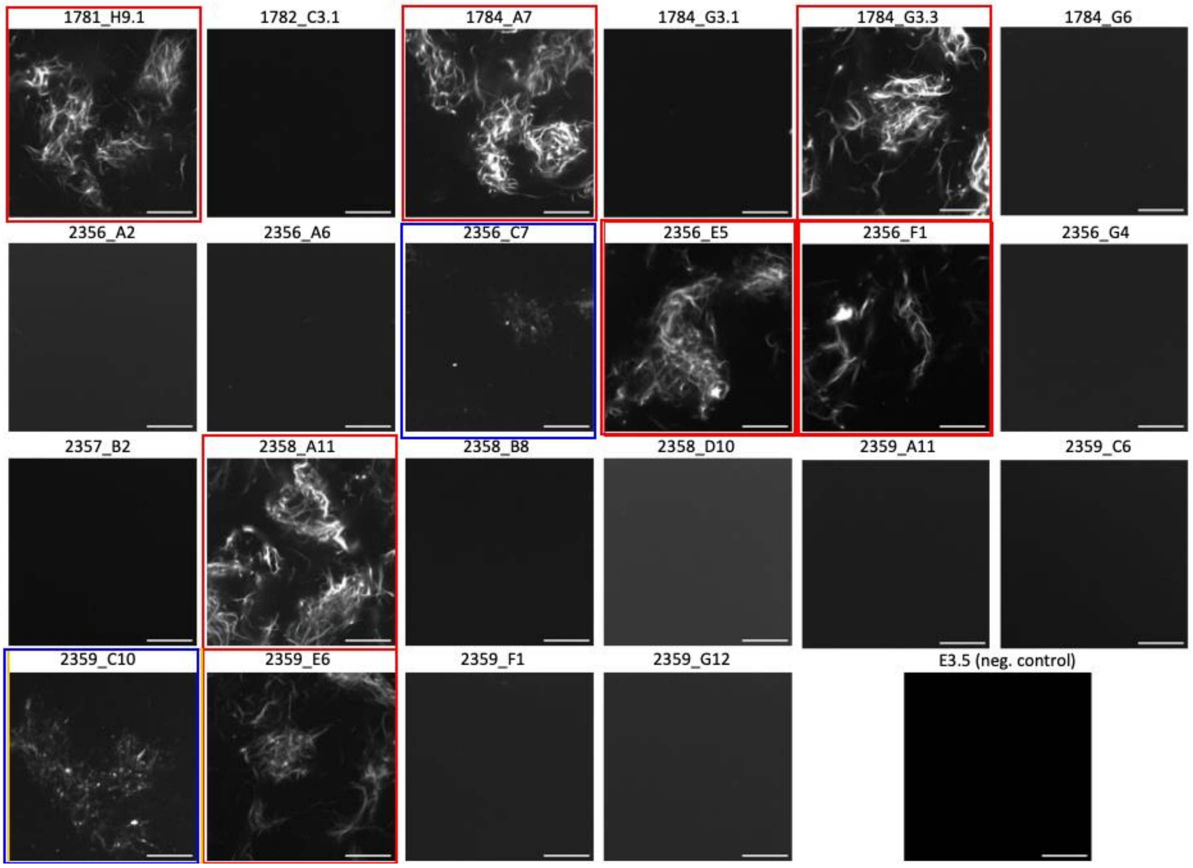

Figure S6. *In vitro* F-actin staining using DARPins. Confocal microscopy images of F-actin stained with His-tagged DARPins and anti-His-tag secondary antibody (mouse IgG_1-FITC). Only 7 out of the 22 tested DARPins could visualize F-actin structures *in vitro* (highlighted in red boxes) and additional two DARPins stained F-actin partially (highlighted in blue boxes). Scale bars: 30 μm

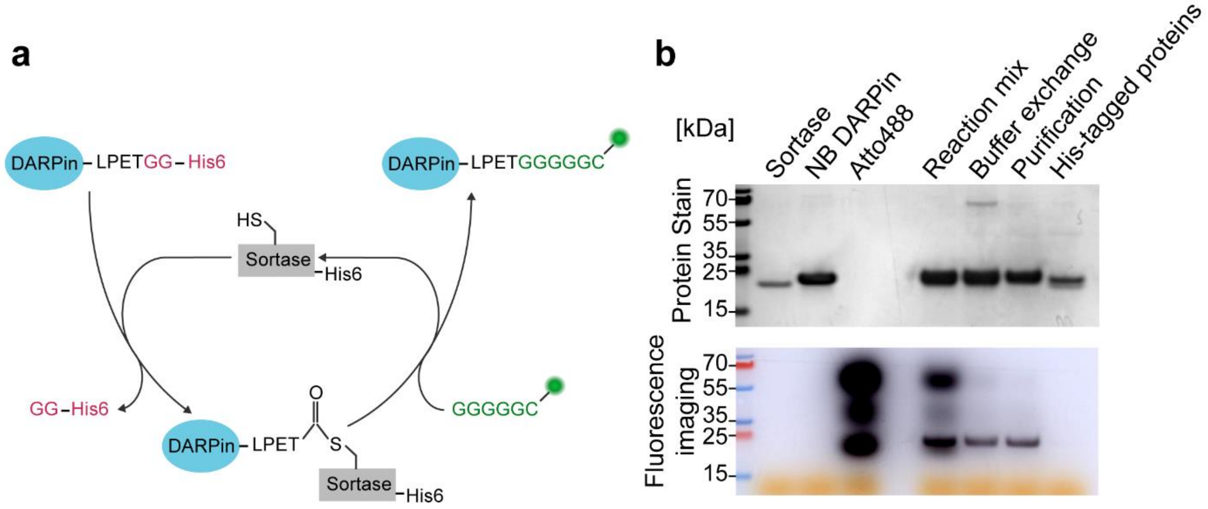

Figure S7. Sortase-mediated labeling of the control DARPin with an Atto488-linked peptide. a. Purified DARPins with a C-terminal sortase recognition sequence (LPETGG) and a His-tag were coupled to a fluorescence-labeled poly-glycine peptide via a sortase-mediated enzymatic reaction. Thereby, the C-terminus after the LPET sequence is replaced with the poly-glycine sequence of a G_5C-peptide that is functionalized with a fluorescent molecule via the thiol group. **b.** SDS-PAGE of the individual components and different steps of the DARPin labelling and purification procedure. The control DARPin is the unselected DARPin (E3_5). “Reaction mix” contains all proteins and labeling peptide. “Buffer exchange” refers to the fraction after unreacted labeling peptides were removed while the reaction buffer was changed to the injection buffer. “Purification” contains the supernatant after incubation with Ni-NTA magnetic beads and was used for microinjections. “His-tagged proteins” contains the remaining sample that was bound to magnetic beads and removed with imidazole.

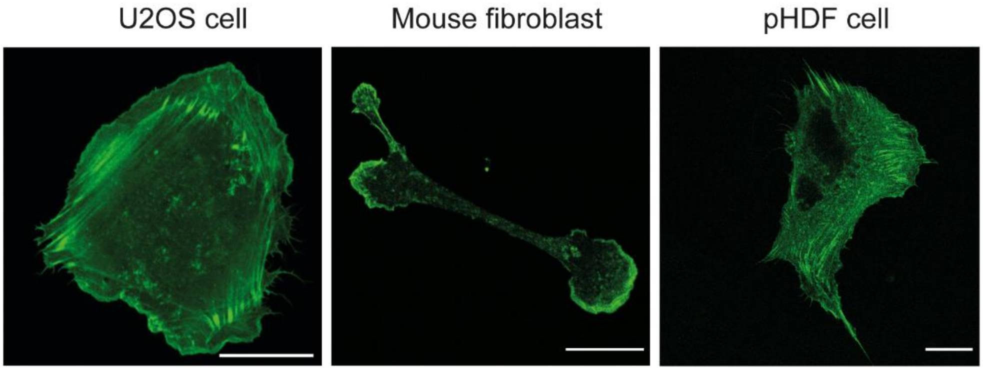

Figure S8. Localization of actin-binding DARPin 1784_A7 in three different mammalian cell lines. Live cells were injected with Cy5-labeled DARPin 1784_A7 12 hours after seeding on glass with different coatings. The U2OS cells were seeded on fibronectin-coated glass, the mouse fibroblasts were seeded on glass and the pHDF cells on vitronectin-coated glass. Confocal microscopy images of live cells were acquired 1-3 hours after microinjection. Scale bar = 20 µm.

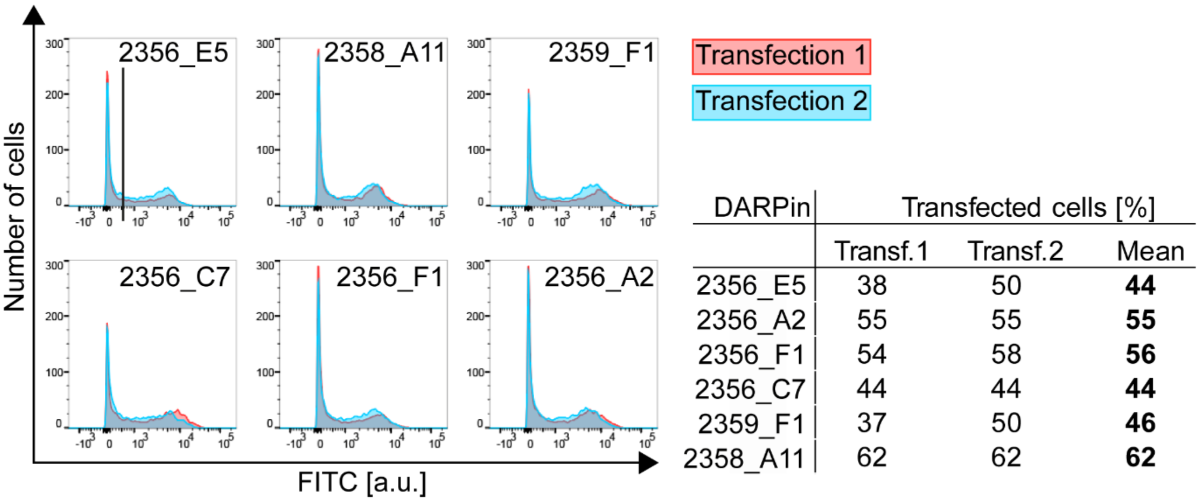

DARPin	Transfected cells [%]		
	Transf.1	Transf.2	Mean
2356_E5	38	50	**44**
2356_A2	55	55	**55**
2356_F1	54	58	**56**
2356_C7	44	44	**44**
2359_F1	37	50	**46**
2358_A11	62	62	**62**

Figure S9. Transfection efficiency of plasmids encoding GFP-actin-DARPins in U2OS cells. The transfection efficiency and expression level of GFP-DARPins was measured 24 hours after transfection by flow cytometry in two independent experiments. The number of transfected cells and the expression levels of the DARPins were similar for all the tested DARPins.

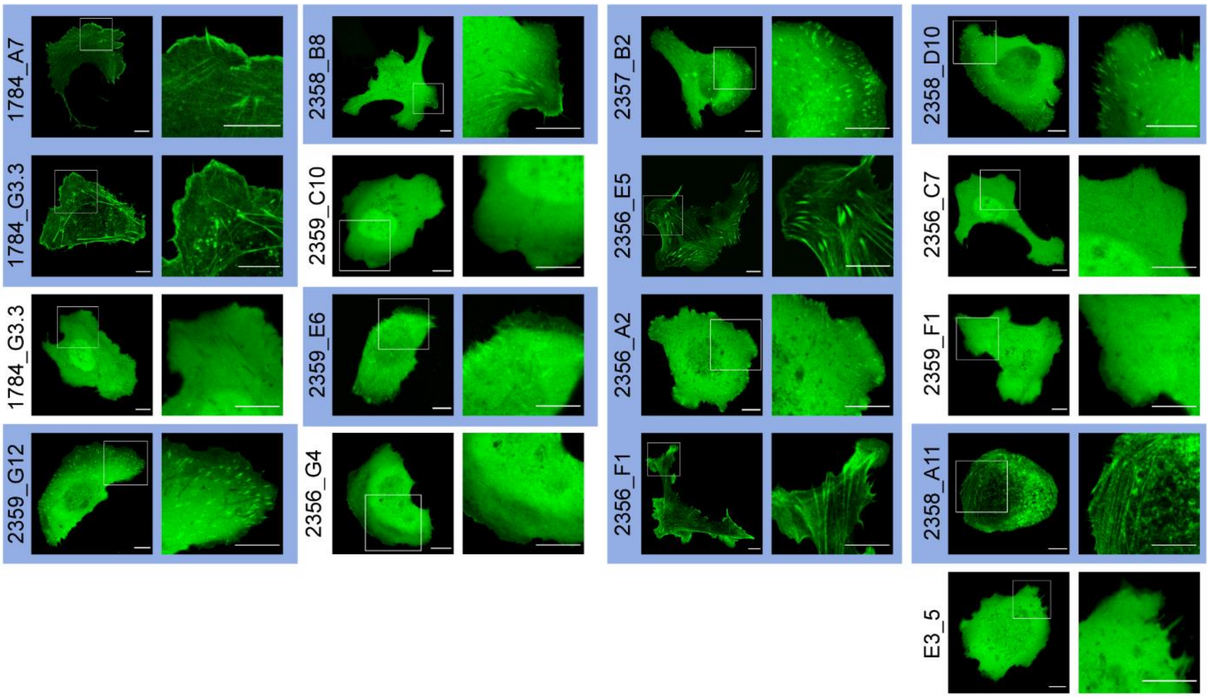

Figure S10. Localization of actin-binding mEGFP-DARPins in living U2OS cells. Confocal microscopy images of live U2OS cells were taken 27-30 hours after transfection with mEGFP-DARPins that were seeded for three hours on fibronectin-coated glass. The non-binder DARPin E3_5 was included as a control. 11 DARPins accumulate in actin-like structures (blue boxes). Scale bars: 10 µm.

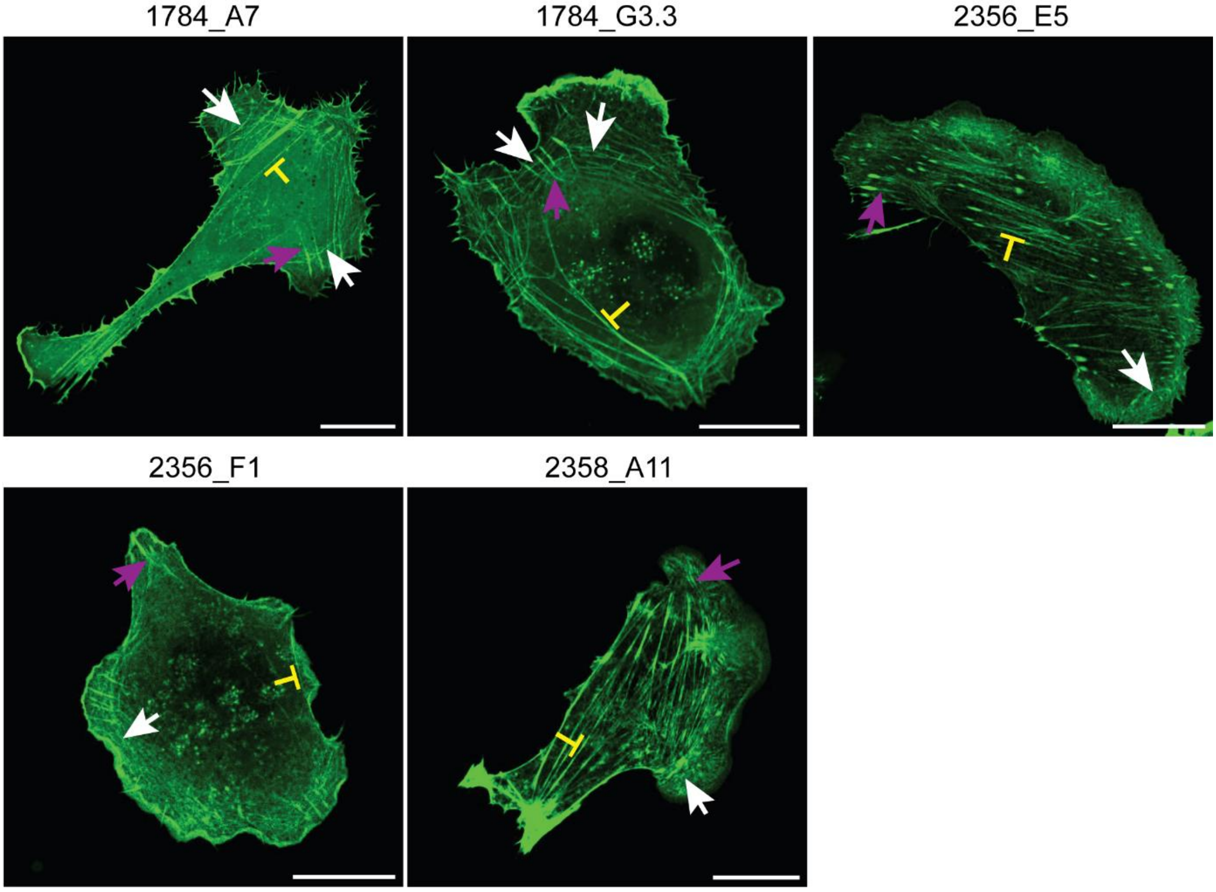

Figure S11. Localization of actin-binding mEGFP-DARPins on ventral and dorsal stress fibers and transverse arcs in living U2OS cells. Confocal microscopy images of live U2OS cells were taken 27-30 hours after transfection with mEGFP-DARPins that were seeded for three hours on fibronectin-coated glass. Stress fibers were categorized in ventral (yellow, blunt-end arrow), dorsal (purple arrow) and transverse arcs (white arrow). Scale bars: 20 µm.

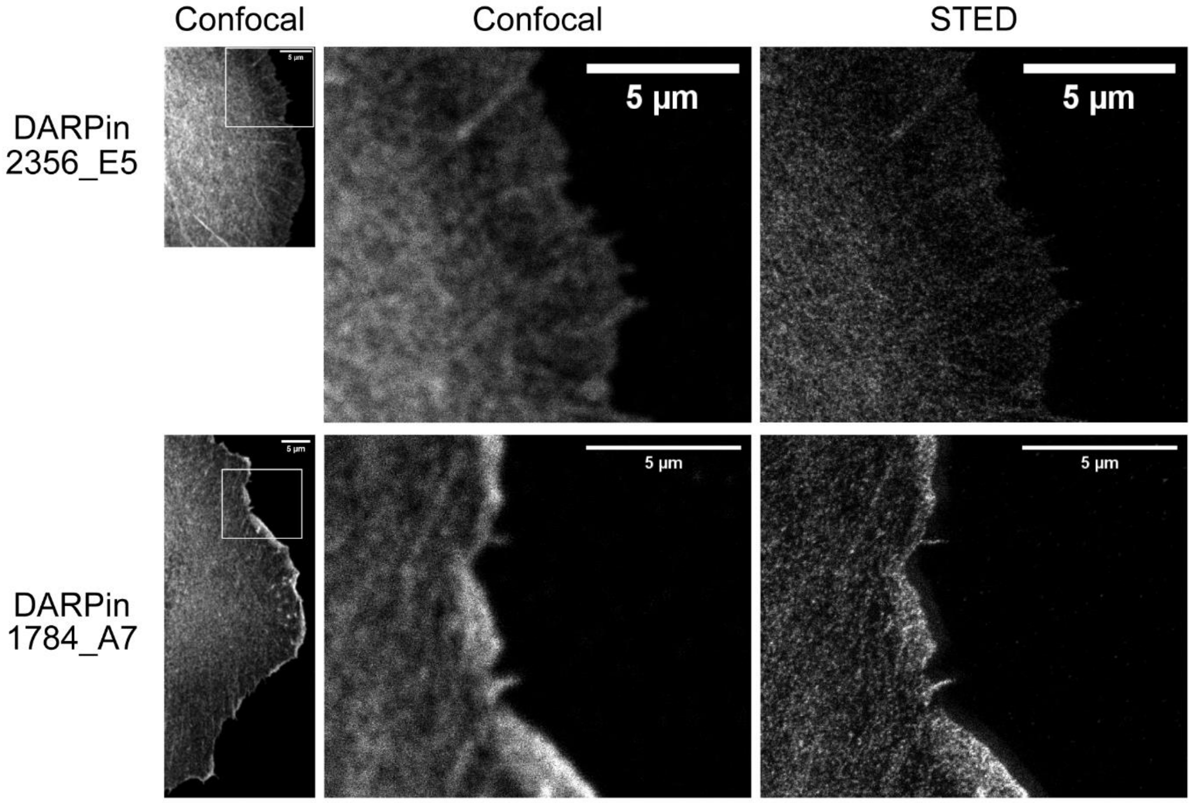

Figure S12. Stimulated emission depletion (STED) imaging of fixed U2OS cells expressing DARPin 1784_A7 and DARPin 2356_E5. U2OS cells transiently transfected with mEGFP-DARPin 1784_A7 or 2356_E5 were fixed and stained with an anti-GFP nanobody coupled to Star635P, which is a suitable fluorophore for STED imaging. The STED images confirm data from confocal imaging on the localization of DARPins and provide higher resolution. DARPin 1784_A7 accumulates at lamellipodia of fixed cells, while DARPin 2356_E5 is not concentrated in the lamellipodium.

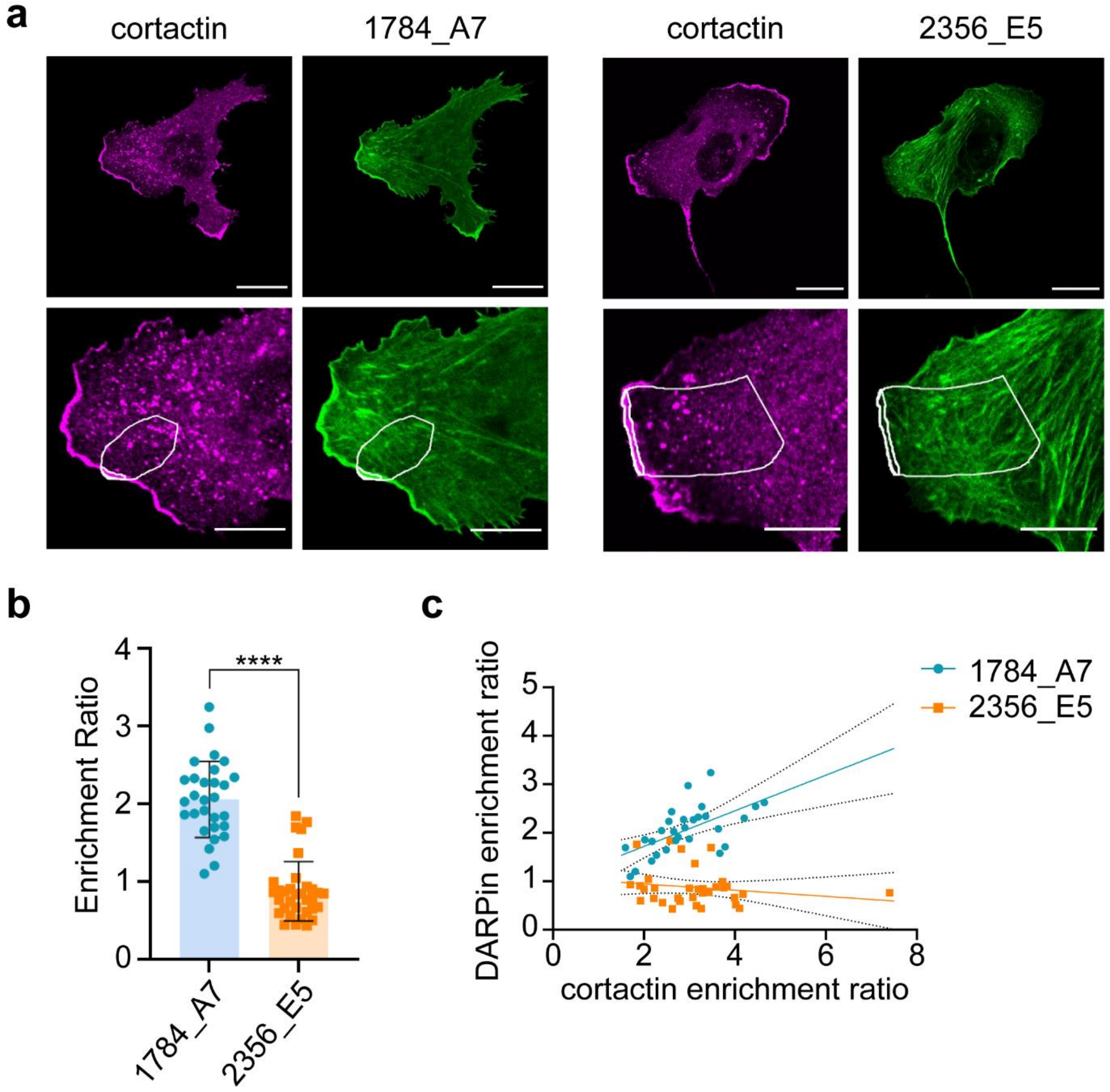

Figure S13. Enrichment of DARPin 1784_A7 in the lamellipodia compared to DARPin 2356_E5. a. The images from Figure 4 of the main text showing U2OS cells transiently transfected with plasmids encoding DARPin 1784_A7 or DARPin 2356_E5 seeded on FN-coated glass and fixed 3 h later were used to exemplify the calculation of the enrichment ratio (ER). The average intensity in the lamellipodium (stained by cortactin) was measured and divided by the average intensity in a region including the lamellipodium and cytoplasm (highlighted in the images). **b.** The enrichment ratios (ER) of DARPin 1784_A7 and DARPin 2356_E5 were measured for n=29 cells and n=33 cells, respectively, in two independent experiments (unpaired t-test, $p<0.0001$). **c.** The enrichment ratios of both DARPins were plotted against the ER of cortactin and a correlation was observed with DARPin 1784_A7 (simple linear regression), but not with DARPin 2356_E5, N=2 experiments.

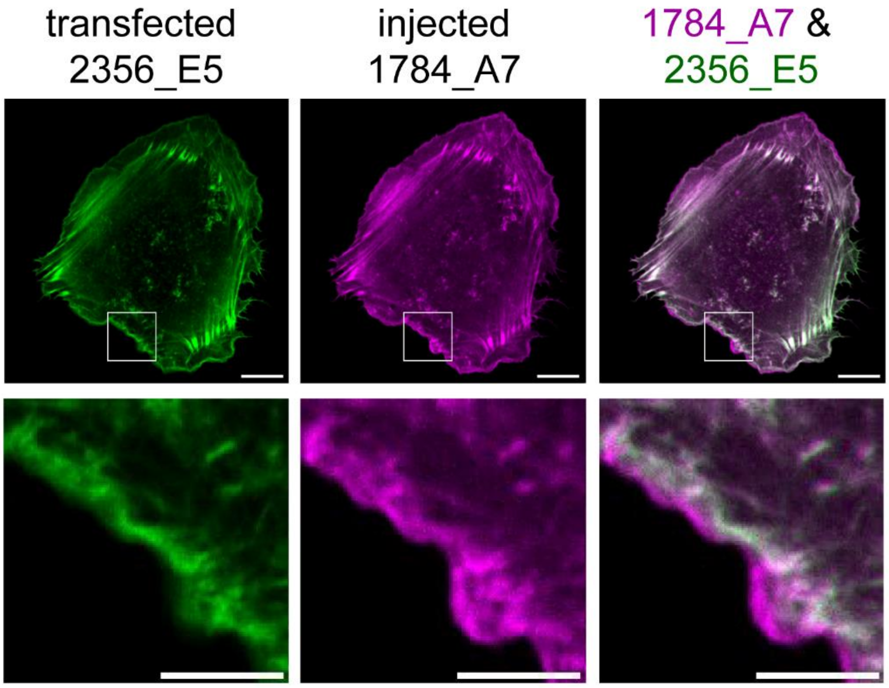

Figure S14. Injection of DARPin 1784_A7 as protein in live cell transfected with DARPin 2356_E5. Cells expressing mEGFP-DARPin 2356_E5 were seeded on FN-coated glass and injected after 3 hours with purified and labelled Cy5-DARPin 1784_A7. The localization patterns of both DARPins remain the same like when being expressed separately. DARPin 1784_A7 accumulated further at the cell edge in lamellipodia while DARPin 2356_E5 remains in the lamella region. Representative image from n=10 cells, N=1 experiment. Scale bars: 10 µm.

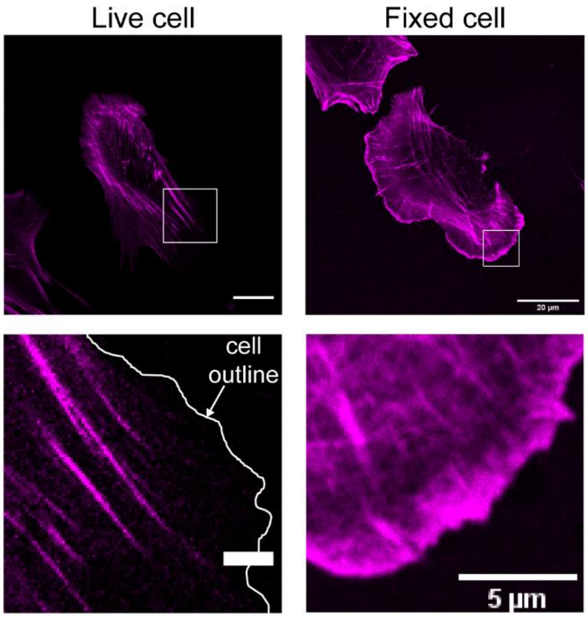

Figure S15. SiR-actin localization in live and fixed U2OS cells. SiR-actin was added to the culture medium of living pHDF cells seeded on fibronectin-coated glass, and confocal microscopy images were acquired 6.5 h after the incubation began (left panels). Confocal microscopy images of U2OS cells seeded on fibronectin-coated glass, fixed with 4% PFA and stained with SiR-actin (right panels). In living cells SiR-actin concentrated on mature stress fibers and was absent from the cell edge (lamellipodia; cortical actin) while in fixed cells SiR-actin efficiently stained both lamellipodia and stress fibers. Scale bars: 20 µm, in details scale bars: 5 µm.

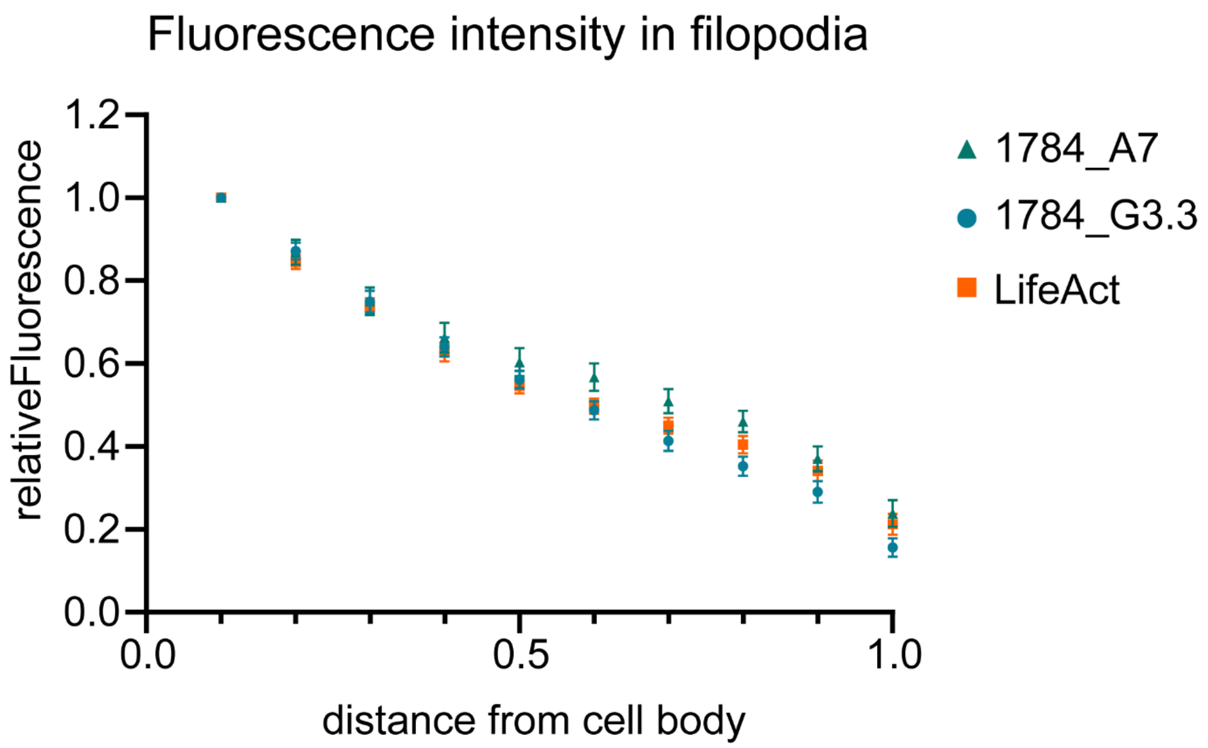

Figure S16. Filopodia labeling in live cells. U2OS cells were transiently co-transfected with mCherry-LifeAct and either mEGFP-DARPin 1784_A7 or 1784_G3.3. Videos of protruding cells were acquired by confocal microscopy. The fluorescence intensity was measured in 10 equal bins along the length of growing filopodia and divided by the average fluorescence intensity in the whole cell. Additionally, the intensity was normalized to the base of the filopodia at the cell body (relative fluorescence). The relative fluorescence of LifeAct and the DARPins was plotted as a function of the distance from the cell body. Mean and SEM for DARPin 1784_A7 n=24 filopodia of 11 cells from two independent experiments, DARPin 1784_G3.3 and LifeAct n=26 filopodia of 10 cells from two independent experiments, LifeAct n=50 filopodia from 21 cells from 4 independent experiments.

Table S1. Amino acids of 16 actin-binding and one control DARPin screened in U2OS cells.

Amino acid sequence of DARPins selected to bind actin. The consensus sequence is shown in bold letters at the top. Randomized amino acids are indicated as “X” and shaded. Point mutations or insertions are also shaded. The control DARPin is abbreviated with NB (non-binder).

N-cap

DARPin	D	L	G	K	K	L	L	E	A	A	R	A	G	Q	D	D	E	V	R	I	L	M	A	N	G	A	D	V	N	A
1784_A7	D	L	G	K	K	L	L	E	A	A	R	A	G	Q	D	D	E	V	R	I	L	M	A	N	G	A	D	V	N	A
1784_G3.3	D	L	G	K	K	L	L	E	A	A	R	A	G	Q	D	D	E	V	R	I	L	M	A	N	G	A	D	V	N	A
2356_E5	D	L	G	K	K	L	L	E	A	A	R	A	G	Q	D	D	E	V	R	I	L	M	A	N	G	A	D	V	N	A
2356_F1	D	L	G	K	K	L	L	E	A	A	R	A	G	Q	D	D	E	V	R	I	L	M	A	N	G	A	D	V	N	A
2358_A11	D	L	G	K	K	L	L	E	A	A	R	A	G	Q	D	D	E	V	R	I	L	M	A	N	G	A	D	V	N	A
2359_G12	D	L	G	K	K	L	L	E	A	A	V	R	G	Q	D	D	E	V	R	I	L	M	A	N	G	A	D	V	N	A
2358_B8	D	L	G	K	K	L	L	E	A	A	L	W	G	H	L	D	E	V	R	I	L	M	A	N	G	A	D	V	N	A
2359_E6	D	L	G	K	K	L	L	E	A	A	R	A	G	Q	D	D	E	V	R	I	L	M	A	N	G	A	D	V	N	A
2357_B2	D	L	G	K	K	L	L	E	A	A	V	R	G	Q	D	D	E	V	R	I	L	M	A	N	G	A	D	V	N	A
2356_A2	D	L	G	K	K	L	L	E	A	A	E	N	G	Q	D	D	E	V	R	I	L	M	A	N	G	A	D	V	N	A
2358_D10	D	L	G	K	K	L	L	E	A	A	E	N	G	Q	D	D	E	V	R	I	L	M	A	N	G	A	D	V	N	A
1784_G3.1	D	L	G	K	K	L	L	E	A	A	R	A	G	Q	D	D	E	V	R	I	L	M	A	N	G	A	D	V	N	A
2359_C10	D	L	G	K	K	L	L	E	A	A	R	A	G	Q	D	D	E	V	R	I	L	M	A	N	G	A	D	V	N	A
2356_G4	D	L	G	K	K	L	L	E	A	A	R	A	G	Q	D	D	E	V	R	I	L	M	A	N	G	A	D	V	N	A
2356_C7	D	L	G	K	K	L	L	E	A	A	R	A	G	Q	D	D	E	V	R	I	L	M	A	N	G	A	D	V	N	A
2359_F1	D	L	G	K	K	L	L	E	A	A	M	F	G	Q	D	D	E	V	R	I	L	M	A	N	G	A	D	V	N	A
NB	D	L	G	K	K	L	L	E	A	A	R	A	G	Q	D	D	E	V	R	I	L	M	A	N	G	A	D	V	N	A

1st repeat

DARPin	X	D	X	X	G	X	T	P	L	H	L	A	A	X	X	G	H	L	E	I	V	E	V	L	L	K	X	G	A	D	V	N	A
1784_A7	V	D	N	R	G	K	T	P	L	H	L	A	A	W	A	G	H	L	E	I	V	E	V	L	L	K	A	G	A	D	V	N	A
1784_G3.3	V	D	E	W	G	R	T	P	L	H	L	A	A	H	K	G	H	L	E	I	V	E	V	L	L	K	T	G	A	D	I	N	A
2356_E5	T	D	W	Y	G	K	T	P	L	H	L	A	A	Y	E	G	H	L	E	I	V	E	V	L	L	K	T	G	A	D	V	N	A
2356_F1	T	D	W	F	G	R	T	P	L	H	L	A	A	S	K	G	H	L	E	I	V	E	V	L	L	K	T	G	A	D	V	N	A
2358_A11	F	D	A	F	G	Y	T	P	L	H	L	A	A	I	Q	G	H	L	E	I	V	E	V	L	L	K	A	G	A	D	V	N	A
2359_G12	I	D	E	Y	G	H	T	P	L	H	L	A	A	E	W	G	H	L	E	I	V	E	V	L	L	K	T	G	A	D	V	N	A
2358_B8	S	D	V	S	G	R	T	P	L	H	L	A	A	A	W	G	H	L	E	I	V	E	V	L	L	K	H	G	A	D	V	N	A
2359_E6	S	D	F	T	G	Y	T	P	L	H	L	A	A	A	W	G	H	L	E	I	V	E	V	L	L	K	T	G	A	D	V	N	A
2357_B2	I	D	E	Y	G	H	T	P	L	H	L	A	A	E	W	G	H	L	E	I	V	E	V	L	L	K	T	G	A	D	V	N	A
2356_A2	S	D	I	Y	G	N	T	P	L	H	L	A	A	W	I	G	H	L	E	I	V	E	V	L	L	K	T	G	A	D	V	N	A
2358_D10	S	D	I	Y	G	N	T	P	L	H	L	A	A	W	I	G	H	L	E	I	V	E	V	L	L	K	T	G	A	D	V	N	A
1784_G3.1	Q	D	S	S	G	F	T	P	L	H	L	A	A	A	V	G	H	L	E	I	V	E	V	L	L	K	T	G	A	D	V	N	A
2359_C10	Q	D	W	M	G	Q	T	P	L	H	L	A	A	W	Q	G	H	L	E	I	V	E	V	L	L	K	T	G	A	D	V	N	A
2356_G4	S	D	L	S	G	W	T	P	L	H	L	A	A	Y	N	G	H	L	E	I	V	E	V	L	L	K	A	G	T	D	V	N	A
2356_C7	I	D	E	W	G	V	T	P	L	H	L	A	A	W	H	G	H	L	E	I	V	E	V	L	L	K	T	S	A	D	V	N	A
2359_F1	T	D	R	F	G	R	T	P	L	H	L	A	A	A	M	G	H	L	E	I	V	E	V	L	L	K	T	G	A	D	V	N	A
NB	T	D	N	D	G	Y	T	P	L	H	L	A	A	S	N	G	H	L	E	I	V	E	V	L	L	K	N	G	A	D	V	N	A

2nd repeat

DARPin	X	D	X	X	G	X	T	P	L	H	L	A	A	X	X	G	H	L	E	I	V	E	V	L	L	K	X	G	A	D	V	N	A
1784_A7	D	D	K	M	G	Y	T	P	L	H	L	A	A	Y	D	G	H	L	E	I	V	E	V	L	L	K	H	G	A	D	V	N	A
1784_G3.3																																	
2356_E5	I	D	Y	F	G	K	T	P	L	H	L	A	A	M	E	G	H	L	E	I	V	E	V	L	L	K	A	G	A	D	V	N	A
2356_F1	V	D	M	R	G	F	T	P	L	H	L	A	A	V	E	G	H	L	E	I	V	E	V	L	L	K	A	G	A	D	V	N	A
2358_A11																																	
2359_G12	Y	D	A	W	G	N	T	P	L	H	L	A	A	W	I	G	H	L	E	I	V	E	V	L	L	K	A	G	A	D	V	N	A
2358_B8																																	
2359_E6	Q	D	M	R	G	S	T	P	L	H	L	A	A	S	E	G	H	L	E	I	V	E	V	L	L	K	A	G	A	D	V	N	A
2357_B2	Y	D	A	W	G	N	T	P	L	H	L	A	A	W	I	G	H	L	E	I	V	E	V	L	L	K	A	G	A	D	V	N	A
2356_A2	K	D	T	F	G	Y	T	P	L	H	L	A	A	D	Y	G	H	L	E	I	V	E	V	L	L	K	H	G	A	D	V	N	A
2358_D10	K	D	T	F	G	Y	T	P	L	H	L	A	A	D	Y	G	H	L	E	I	V	E	V	L	L	K	T	G	A	D	V	N	A
1784_G3.1	Q	D	I	D	G	D	T	P	L	H	L	A	A	V	L	G	H	L	E	I	V	E	V	L	L	K	H	G	A	D	V	N	A
2359_C10	I	D	D	I	G	A	T	P	L	H	L	A	A	V	V	G	H	L	E	I	V	E	V	L	L	K	A	G	A	D	V	N	A
2356_G4	I	D	W	I	G	K	T	P	L	H	L	A	A	Y	E	G	H	L	E	I	V	E	V	L	L	K	H	G	A	D	V	N	A
2356_C7	A	D	W	Q	G	M	T	P	L	H	L	A	A	W	V	G	H	L	E	I	V	E	V	L	L	K	H	G	A	D	V	N	A
2359_F1																																	
NB	S	D	L	T	G	I	T	P	L	H	L	A	A	A	T	G	H	L	E	I	V	E	V	L	L	K	H	G	A	D	V	N	A

3rd repeat

DARPin	X	D	X	X		G	X	T	P	L	H	L	A	A	X	X	G	H	L	E	I	V	E	V	L	L	K	X	G	A	D	V	N	A
1784_A7																																		
1784_G3.3																																		
2356_E5	Q	D	N	L		G	Y	T	P	L	H	L	A	A	Y	N	G	H	L	E	I	V	E	V	L	L	K	H	G	A	D	V	N	A
2356_F1	Q	D	N	L		G	Y	T	P	L	H	L	A	A	Y	N	G	H	L	E	I	V	E	V	L	L	K	H	G	A	D	V	N	A
2358_A11																																		
2359_G12	L	D	M	R		G	Y	T	P	L	H	L	A	A	D	Y	G	H	L	E	I	V	E	V	L	L	K	H	G	A	D	V	N	A
2358_B8																																		
2359_E6	F	D	W	H		G	F	T	P	L	H	L	A	A	H	Y	G	H	L	E	I	V	E	V	L	L	K	H	G	A	D	V	N	A
2357_B2	L	D	M	R		G	Y	T	P	L	H	L	A	A	D	Y	G	H	L	E	I	V	E	V	L	L	K	H	G	A	D	V	N	A
2356_A2																																		
2358_D10	V	D	R	L	N	G	H	T	P	L	H	L	A	A	K	V	G	H	L	E	I	V	E	V	L	L	K	H	G	A	D	V	N	A
1784_G3.1	K	D	S	D		G	H	T	P	L	H	L	A	A	W	I	G	H	L	E	I	V	E	V	L	L	K	H	G	A	D	V	N	A
2359_C10	M	D	Q	A		G	W	T	P	L	H	L	A	A	F	R	G	H	L	E	I	V	E	V	L	L	K	H	G	A	D	V	N	A
2356_G4																																		
2356_C7	I	D	D	S		G	L	T	P	L	H	L	A	A	H	D	G	H	L	E	I	V	E	V	L	L	K	H	G	A	D	V	N	A
2359_F1																																		
NB	Y	D	N	D		G	H	T	P	L	H	L	A	A	K	Y	G	H	L	E	I	V	E	V	L	L	K	H	G	A	D	V	N	A

C-cap

DARPin	Q	D	K	F	G	K	T	P	F	D	L	A	I	D	N	G	N	E	D	I	A	E	V	L	Q	K	A	A
1784_A7	Q	D	K	F	G	K	T	P	F	D	L	A	I	D	N	G	N	E	D	I	A	E	V	L	Q	K	A	A
1784_G3.3	T	D	Q	I	G	R	T	P	L	H	L	A	A	T	W	G	H	L	E	I	V	E	V	L	Q	K	A	A
2356_E5	Q	D	K	F	G	K	T	P	F	D	L	A	I	D	N	G	N	E	D	I	A	E	V	L	Q	K	A	A
2356_F1	Q	D	K	F	G	K	T	P	F	D	L	A	I	D	N	G	N	E	D	I	A	E	V	L	Q	K	A	A
2358_A11	Q	D	I	I	G	R	T	P	F	D	L	A	A	W	N	G	N	E	D	I	A	E	V	L	Q	K	A	A
2359_G12	Q	D	K	F	G	K	T	P	F	D	L	A	I	D	N	G	N	E	D	I	A	E	V	L	Q	K	A	A
2358_B8	Q	D	K	Y	G	W	T	P	F	D	L	A	I	D	N	G	N	E	D	I	A	E	V	L	Q	K	A	A
2359_E6	Q	D	K	F	G	K	T	P	F	D	L	A	I	D	N	G	N	E	D	I	A	E	V	L	Q	K	A	A
2357_B2	Q	D	K	F	G	K	T	A	F	D	I	S	I	D	N	G	N	E	D	I	A	E	V	L	Q	K	A	A
2356_A2	Q	D	Y	Y	G	Q	T	A	F	D	I	S	I	D	N	G	N	E	D	I	A	E	V	L	Q	K	A	A
2358_D10	Q	D	K	F	G	K	T	P	F	D	L	A	A	D	D	G	N	E	D	I	A	E	V	L	Q	K	A	A
1784_G3.1	Q	D	K	F	G	K	T	P	F	D	L	A	I	D	N	G	N	E	D	I	A	E	V	L	Q	K	A	A
2359_C10	Q	D	K	F	G	K	T	P	F	D	L	A	I	D	N	G	N	E	D	I	A	E	V	L	Q	K	A	A
2356_G4	Q	D	K	F	G	K	T	P	F	D	L	A	I	D	N	G	N	E	D	I	A	E	V	L	Q	K	A	A
2356_C7	Q	D	K	F	G	K	T	P	F	D	L	A	I	D	N	G	N	E	D	I	A	E	V	L	Q	K	A	A
2359_F1	A	D	K	Y	G	W	T	P	F	D	L	A	I	D	N	G	N	E	D	I	A	E	V	L	Q	K	A	A
NB	Q	D	K	F	G	K	T	A	F	D	I	S	I	D	N	G	N	E	D	L	A	E	I	L	Q			

Table S2. Intracellular localization of actin-binding DARPins. The localization of mEGFP-labeled DARPins was qualitatively assessed in live U2OS cells via confocal fluorescence imaging.

DARPin	stress fibers	lamellipodia	focal adhesions	cytoplasmic	nuclear
1784_A7	x	x	x		
1784_G3.3	x	x	x		
1784_G3.1				x	x
2359_G12			x	x	
2358_B8			x	x	
2359_C10				x	x
2359_E6			x	x	
2357_B2			x	x	
2356_E5	x		x		
2356_A2			x	x	
2356_F1	x		x		
2356_G4				x	
2358_D10			x	x	
2356_C7				x	x
2359_F1				x	
2358_A11	x		x		

Table S3: Reagents used as purchased.

Reagent	Abbreviation	Supplier	Cat. No.
**Globular actin**	G-actin	Cytoskeleton Inc.	AKL99
**Biotinylated actin**	G-actin-biotin	Cytoskeleton Inc.	AB07
**Rhodamine-labeled actin**	G-actin-Rhod	Cytoskeleton Inc.	AR05
**Pyrene-labeled actin**	G-actin-pyrene	Hypermol	8202-01
**Bovine serum albumin**	BSA	Sigma	A7030-1KG
**Fibronectin**	FN	Sigma	F1141-1MG
**SiR-actin**		Tebu-bio	251SC001
**Tetramethylrhodamine-isothiocyanate-Phalloidin**	TRITC-Phalloidin	Sigma	P1951
**Triton-X100**		Sigma	T8787-100ML
**Opti-MEM ® I 1x**		Gibco	31985-070
**Lipofectamine 3000**		Invitrogen	L3000-008
**0.05% trypsin-EDTA**		ThermoScientific	25300062
**McCoy5A**		ATCC	30-2007
**CO_2 independent medium**		Gibco	18045-054
**Fetal bovine serum**	FBS	ThermoScientific	10500064
**Penicillin-Streptomycin (10000 U/ml)**	PenStrep	ThermoScientific	15140122
**Ampicillin**	Amp	Sigma	A9518
**Kanamycin**	Kan	Carl Roth	T832.2
**DPBS 1x**		Gibco	14190-094
**Sodium Pyruvat (100 mM)**		Gibco	11360-070
**Paraformaldehyde**	PFA	Carl Roth	0335.1
***E.coli* BL21DE3**		ThermoScientific	EC0114
***E.coli* DH5α**		ThermoScientific	18265017
***E.coli* XL-1 blue**		Stratagene; now available from Agilent	200249
**Fibronectin bovine plasma**		Sigma	F1141-1MG
**$CaCl_2$**		Roth	A119.1
**NaCl**		Emsure	1.06404.1000
**Tris-HCl**		Biomol	8015,1
**K_2HPO_4**		Emsure	1.05104.1000
**KH_2PO_4**		Emsure	1.04873.1000
**$NaH_2PO_4*H_2O$**		Emsure	1.06346.1000
**imidazole**		Sigma	I5513
**NH_4Cl**		Sigma	A4514-500G
**Glycin**		Riedel de Haen	332226
**HEPES**		Carl Roth	9105.3
**KCl**		Carl Roth	6781.3
**$MgCl_2$**		Carl Roth	2189.2

**PlusOne Mini Dialysis Kit MWCO 8K, 2ml**		GE Healthcare	28955965
**Isopropyl-β-D-thiogalactosid**	IPTG	Roche	10724815001
**CelLytic B 10x**		Sigma	C8740
**Lysozyme**		Sigma	62971
**Pierce Universal Nuclease**		ThermoScientific	88701
**Dithiothreitol**	DTT	Serva	20710
**Adenosin-5'-triphosphate.Na_2-salt**	ATP	Serva	10920
**Glycerol**		Sigma	G9012
**MES SDS running buffer (20x)**		Novex	B0002
**InstantBlue coomassie stain**		Abcam	ISB1L
**Y-27632 dihydrochloride**		Enzo	ALX-270-333-M001
**Jasplakinolide**		Abcam	ab141409
**Latranculin B actin polymerization inhibitor**		Abcam	ab144291
**B-Per Direct detergent**		ThermoScientific	78248
**Streptavidin-Tb cryptate**		Cisbio	610SATLB
**Tag-lite assay buffer**		Cisbio	

Table S4. Antibodies used in our study.

Antibody	Clone	Application / Dilution	Supplier	Cat. No.
**anti-FLAG RGS(His)$_4$ IgG (mouse)**	M2	ELISA/1:5000	Sigma-Aldrich	F3165
**anti-FLAG RGS(His)$_4$ IgG (mouse) d2 conjugate**	M2	HTRF	Cisbio	61FG2DLB
**anti-mouse IgG (goat) alkaline phosphatase conjugate**	polyclonal	ELISA/1:10.000	Sigma-Aldrich	A3562
**anti-His$_6$-tag IgG FITC conjugate**	AD1.1.10	In solution staining /1:5,6	Thermo-Fisher	MA1-81891
**anti-cortactin IgG**	H-191	Immunofluorescence / 1:150	Santa-Cruz	SC-11408
**anti-mouse IgG AlexaFluor488 conjugate**	polyclonal	Immunofluorescence / 1:150	Thermo-Fisher	A11001
**Anti-paxillin IgG**	Y113	Immunofluorescence/ 1:100	Abcam	Ab32084
**anti-rabbit IgG AlexaFluor568 conjugate**	polyclonal	Immunofluorescence / 1:150	Thermo-Fisher	A21238
**anti-rabbit IgG AlexaFluor647 conjugate**	polyclonal	Immunofluorescence / 1:150	Thermo-Fisher	A21244
**Nanobody Fluotagx4GFP coupled with Star635P**	NanoTag Biotechnologies	Immunofluorescence for STED/ 1:250	Nanobody Fluotagx4GFP coupled with Star635P	N0304

Movie S1. Time-lapse, live-cell fluorescence confocal microscopy imaging of human dermal fibroblasts (pHDF) microinjected with fluorescent DARPin 1784_A7 and subsequently incubated with SiR-actin. DARPin (left panel, green) accumulated in lamellipodia and stress fibers, while SiR-actin (middle panel, magenta) accumulated mostly in mature stress fibers and only partially overlapped with DARPin. Right panel shows the merged channels. Scale bar: 20 µm.

Movie S2. Time-lapse live-cell fluorescence confocal microscopy imaging of U2OS transiently transfected with mEGFP-DARPin 1784_A7. Cells were seeded for 2 hours on soft silicone elastomers, pre-coated with fibronectin. Scale bars: 10 µm.

Movie S3. Time-lapse live-cell fluorescence confocal microscopy imaging of U2OS transiently transfected with mEGFP-DARPin 2356_E5. Cells were seeded for 3 hours on soft silicone elastomers, pre-coated with fibronectin. Scale bars: 10 µm.

Movie S4. Time-lapse live-cell fluorescence confocal microscopy imaging (inverted) of U2OS transiently transfected with mEGFP-DARPin 1784_A7. Cells were seeded on fibronectin-coated glass substrates and imaged with a frame rate of 12 frames/minute. Scale bar: 10 µm.

Movie S5. Time-lapse live-cell fluorescence confocal microscopy imaging of U2OS cells transiently transfected with mEGFP-DARPin 2356_E5. Time-lapse imaging was acquired 2-5 min after cells were treated with the JLY drug cocktail arresting the cytoskeleton while remaining alive. DARPin 2356_E5 is localized in the lamellipodia region. Scale bar: 20 µm.

Movie S6. Time-lapse live-cell fluorescence spinning disk confocal microscopy imaging of stable-bleb forming U2OS cells transiently transfected with mEGFP-DARPins or mCherry LifeAct. Time-lapse imaging was acquired 30 min to 3 hours after cells were confined. From left to right cells are expressing mCherry-LifeAct, mEGFP-DARPin 1784_A7, 1784_G3.3, 2356_E5. Scale bar: 20 µm.
